# Molecular determinants for recognition of serotonylated chromatin

**DOI:** 10.1093/nar/gkaf612

**Published:** 2025-07-09

**Authors:** Laura Pulido-Cortés, Hajo Gielingh, Vito Thijssen, Minglong Liu, Ryoji Yoshisada, Leonardo Romão Soares, Sheikh Nizamuddin, Florian Friedrich, Holger Greschik, Ling Peng, Rodrigo Vargas Honorato, Manfred Jung, Alexandre M J J Bonvin, Martin L Biniossek, Roland Schüle, Seino Jongkees, Hugo van Ingen, H Th Marc Timmers

**Affiliations:** German Cancer Consortium (DKTK), partner site Freiburg, a partnership between the DKFZ and Medical Center-University of Freiburg and Department of Urology, Medical Center-University of Freiburg, 79106 Freiburg, Germany; NMR Spectroscopy, Bijvoet Center for Biomolecular Research, Utrecht University, 3584 CH Utrecht, The Netherlands; Department of Chemistry and Pharmaceutical Sciences, Amsterdam Institute of Molecular and Life Sciences, Vrije Universiteit Amsterdam, 1081 HZ Amsterdam, The Netherlands; Chemical Biology and Drug Discovery, Utrecht Institute for Pharmaceutical Sciences, Utrecht University, 3584 CG Utrecht, The Netherlands; Department of Chemistry and Pharmaceutical Sciences, Amsterdam Institute of Molecular and Life Sciences, Vrije Universiteit Amsterdam, 1081 HZ Amsterdam, The Netherlands; Chemical Biology and Drug Discovery, Utrecht Institute for Pharmaceutical Sciences, Utrecht University, 3584 CG Utrecht, The Netherlands; Department of Chemistry and Pharmaceutical Sciences, Amsterdam Institute of Molecular and Life Sciences, Vrije Universiteit Amsterdam, 1081 HZ Amsterdam, The Netherlands; Department of Chemistry and Pharmaceutical Sciences, Amsterdam Institute of Molecular and Life Sciences, Vrije Universiteit Amsterdam, 1081 HZ Amsterdam, The Netherlands; German Cancer Consortium (DKTK), partner site Freiburg, a partnership between the DKFZ and Medical Center-University of Freiburg and Department of Urology, Medical Center-University of Freiburg, 79106 Freiburg, Germany; Institute of Pharmaceutical Sciences, University of Freiburg, 79104 Freiburg, Germany; German Cancer Consortium (DKTK), partner site Freiburg, a partnership between the DKFZ and Medical Center-University of Freiburg and Department of Urology, Medical Center-University of Freiburg, 79106 Freiburg, Germany; German Cancer Consortium (DKTK), partner site Freiburg, a partnership between the DKFZ and Medical Center-University of Freiburg and Department of Urology, Medical Center-University of Freiburg, 79106 Freiburg, Germany; NMR Spectroscopy, Bijvoet Center for Biomolecular Research, Utrecht University, 3584 CH Utrecht, The Netherlands; Institute of Pharmaceutical Sciences, University of Freiburg, 79104 Freiburg, Germany; German Cancer Consortium (DKTK), partner site Freiburg, a partnership between the DKFZ and Medical Center-University of Freiburg, 79106 Freiburg, Germany; NMR Spectroscopy, Bijvoet Center for Biomolecular Research, Utrecht University, 3584 CH Utrecht, The Netherlands; Institute of Molecular Medicine and Cell Research, Medical Center-University of Freiburg, 79016 Freiburg, Germany; German Cancer Consortium (DKTK), partner site Freiburg, a partnership between the DKFZ and Medical Center-University of Freiburg and Department of Urology, Medical Center-University of Freiburg, 79106 Freiburg, Germany; Department of Chemistry and Pharmaceutical Sciences, Amsterdam Institute of Molecular and Life Sciences, Vrije Universiteit Amsterdam, 1081 HZ Amsterdam, The Netherlands; Chemical Biology and Drug Discovery, Utrecht Institute for Pharmaceutical Sciences, Utrecht University, 3584 CG Utrecht, The Netherlands; NMR Spectroscopy, Bijvoet Center for Biomolecular Research, Utrecht University, 3584 CH Utrecht, The Netherlands; German Cancer Consortium (DKTK), partner site Freiburg, a partnership between the DKFZ and Medical Center-University of Freiburg and Department of Urology, Medical Center-University of Freiburg, 79106 Freiburg, Germany

## Abstract

Post-translational modifications of histone tails constitute a key epigenetic mechanism controlling chromatin environment and gene transcription. Serotonylation of histone H3Q5 (H3Q5ser) is a recently discovered mark associated with active transcription of RNA polymerase II (pol II)-transcribed genes. The direct link between H3Q5ser and the pol II transcription machinery relies on the TFIID subunit TAF3. The presence of H3Q5ser enhances TAF3 binding to H3K4me3, but the molecular determinants underlying this interaction remained unclear. Here, we resolve the binding mode of TAF3-PHD to H3K4me3Q5ser identifying a novel binding surface for H3Q5ser using solution nuclear magnetic resonance spectroscopy. This reveals how H3Q5ser recognizes a conserved surface of the TAF3-PHD via CH–π interactions in an edge–face conformation involving a proline residue stabilized by a tryptophan. This combination of proline and tryptophan is unique to the PHD finger of TAF3 and conserved among TAF3 orthologues. Our findings establish a framework for the molecular recognition of serotonylated chromatin, laying the foundation for developing epigenetic inhibitors targeting serotonylation-dependent transcriptional regulation in neuronal development.

## Graphical Abstract

**Graphical Abstract:**
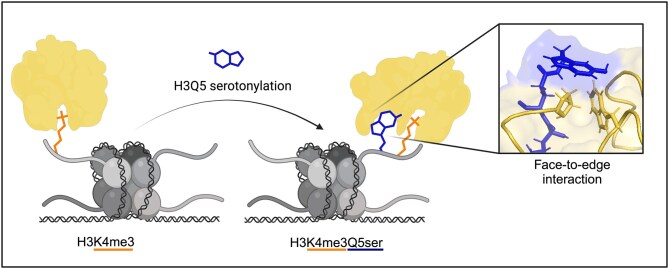

## Introduction

Control of gene transcription by RNA polymerase II (pol II) in eukaryotic genomes is tightly linked to chromatin states. DNA accessibility of regulatory elements such as core promoters or enhancers together with post-translational modifications (PTMs) of histones associated with these elements controls the assembly of productive pol II transcription complexes [1–6]. In this respect, methylation of histone H3 at lysine 4 is of particular interest [7, 8]. Monomethylation (H3K4me1) is associated with enhancers, while trimethylation (H3K4me3) of the +1 nucleosome is a feature of active pol II promoters [3, 7, 8]. The levels of H3K4 methylation are regulated by the SET1/MLL family of methyltransferases and histone demethylases of the KDM5 and KDM1 families [7, 8]. Due to its implication in controlling gene expression, dysregulation of the pathways governing H3K4 methylation contributes to aberrant transcriptional programs associated with human diseases, including cancer and developmental disorders [7–10].

Recent studies revealed that the adjacent glutamine 5 residue (H3Q5) can be modified with the covalent addition of bioactive monoamines by transglutaminase 2 (TGM2) [11–16]. There are three described H3Q5 monoaminylation modifications: H3Q5 serotonylation (H3Q5ser) [11], dopaminylation (H3Q5dop) [12], and histaminylation (H3Q5his) [13]. TGM2-mediated H3Q5 monoaminylation is reversible and can occur on both non-methylated H3 and trimethylated H3K4 tails [11–13]. H3Q5 modifications levels are TGM2-controlled in a cellular context and recent studies link them to a variety of processes, including neurodevelopment, adult brain plasticity, addictive behaviour, placental function, circadian rhythm, or cancer progression [11–22]. H3K4me3Q5ser has been identified across various organisms and tissues, with a preference for the brain, gut, and testis [11]. This combinatorial H3K4me3Q5ser modification is enriched near active TSS regions and regulates gene expression [11, 17–21]. H3Q5 monoaminylation can affect H3K4 methylation control depending on the tolerance or inhibition of H3K4 methylases and demethylases for H3Q5 modifications. In *in vitro* assays H3Q5ser is neutral for the MLL1 H3K4 methyltransferase, but it impairs H3K4me3 demethylation by KDM5B and KDM1A [23].

The connection between modified histone tails and the transcriptional machinery occurs through the recognition of histone PTMs via protein domains like chromodomains, Tudor domains, WD40 domains, and plant homeodomain (PHD) fingers [7, 8]. The PHD finger is a type of zinc finger originally identified in plants as a characteristic cysteine-rich region N-terminal to the homeodomain [24, 25]. PHDs consist of a canonical Cys4–His–Cys3 motif, which coordinates two zinc ions to form a double-stranded antiparallel β-sheet flanked by α-helices [26–32]. This chromatin reader domain is present in many epigenetic regulators and across the eukaryotic kingdom [33, 34].

An important link between H3K4me3 and pol II is formed by the basal transcription factor TFIID [32, 35–37]. TFIID is able to engage with active promoters by DNA contacts as well as by two histone-binding domains: the double bromodomains of TAF1 engaging acylated histones [38, 39] and the TAF3-PHD finger binding H3K4me3 [32, 35–37]. Structural and mutational analyses revealed that the TAF3-PHD engages with H3K4me3 via an aromatic cage, while additional interactions with the H3 N-terminus and H3R2 side chain contribute to specificity for the H3K4 methylation state [32]. Binding of the TAF3-PHD finger to H3K4me3 is among the strongest reported (*K*_D_ = 0.16–0.7 μM) [23, 32, 35, 40] and it is negatively affected by H3R2 asymmetric methylation [32, 35]. Phosphorylation of H3T3 (H3T3ph) abolishes H3K4me3 binding by the TAF3-PHD [41, 42]. Interestingly, H3Q5ser is the first modification leading to an enhanced TAF3-PHD binding to H3K4me3. H3K4me3Q5ser displayed an enhanced TFIID interaction compared to its non-serotonylated counterpart using HeLa cells nuclear extracts [11]. Additionally, investigation of H3Q5ser tolerance by 15 representative H3K4me3 binders revealed slight variations in binding affinity from two-fold to nine-fold in either direction. The TAF3-PHD displays the strongest increase in affinity to H3K4me3 upon H3Q5 serotonylation [23]. By enhancing engagement to TFIID, H3K4me3Q5ser has been linked to the activation of neuronal-specific transcriptional programs upon differentiation [11].

Despite their importance, the molecular details underlying how recognition of the combination of H3K4me3 and H3Q5ser enhances TAF3-PHD binding have remained obscure. In this study, we utilize a combination of solution nuclear magnetic resonance (NMR), modelling, mutagenesis, and affinity measurements to reveal the structure and to identify the molecular determinants underlying the TAF3-PHD preference for H3Q5ser. We find that a unique proline–tryptophan pair of TAF3 is essential for H3Q5ser binding.

## Materials and methods

### Protein expression and purification

#### GST-tagged PHD domains

The coding sequences of *Mus musculus* Taf3-PHD domain (residues 857–924) and *Homosapiens* PHF2 (1–66), PHF8 (37–102), and BPTF(2) (2865–2921) were cloned into the pGEX2T-derived vector, pRP265NB, for bacterial expression of GST-tagged proteins [32, 35]. Note that the PHD fingers of *M. musculus* Taf3 and *H. sapiens* TAF3 differ in a single amino acid. A901 in mouse corresponds to T899 in human. Here, amino acid numbering corresponds to mouse Taf3 isoform 1 with UniProt ID: Q5HZG4, accessible at https://www.uniprot.org/uniprotkb/Q5HZG4/entry. Single or double mutations were introduced by mutagenesis polymerase chain reaction and validated by Sanger sequencing. A single colony of freshly transformed BL21(DE3) *Escherichia coli* bacteria with the corresponding construct was grown in Luria broth (LB) medium supplemented with 50 μg/ml ampicillin until an OD_600_ of 0.7. Protein expression was induced by 0.4 mM isopropyl β-d-thiogalactopyranoside at 18°C overnight. Cell pellets were resuspended in lysis buffer [20 mM Tris–HCl, pH 8.0, 300 mM NaCl, 1× cOmplete™ Protease Inhibitor Cocktail (Roche)] disrupted with an EmulsiFlex French press (Avestin) and lysates were clarified via centrifugation at 130 000 × *g* for 90 min at 4°C. Supernatant was loaded on a self-packed Glutathione Sepharose™ 4 Fast Flow (Amersham Pharmacia Biotech) column, washed extensively with wash buffer (20 mM Tris–HCl, pH 8.0, 100 mM NaCl, 1 mM DTT), and eluted with 50 mM l-reduced glutathione in wash buffer. The eluted protein was further purified in a Mono Q™ 10/100 GL (GE Healthcare) anion exchange column developed by a linear gradient of 100–1000 mM NaCl in 20 mM Tris–HCl (pH 8.0). After concentration, the protein sample was resolved on a Superdex-75 HiLoad 16/600 gel filtration column (GE Healthcare) in isothermal titration calorimetry (ITC) buffer (100 mM NaCl, 20 mM Tris–HCl, pH 7.5). Finally, 30 μM samples were aliquoted, snap-frozen in liquid nitrogen, and stored at −80°C (Supplementary Fig. S1).

#### Purification of ^13^C- and ^15^N-labelled Taf3-PHD for NMR

The ^13^C- and ^15^N-labelled Taf3-PHD NMR samples were purified as described above, with minor differences. A single colony of freshly transformed BL21(DE3) *E. coli* was grown in a synthetic M9 minimal medium (6.0 g/l Na_2_HPO_4_·2H_2_O, 3.0 g/l KH_2_PO_4_, 0.5 g/l NaCl, 25 mg/l MgSO_4_, 290 μg/l CaCl_2_, 1 μg/l FeSO_4_·7H_2_O, 270 μg/l ZnCl_2_, and 5 mg/l thiamine) containing 2.0 g/l ^13^C-glucose as the only carbon source and 0.5 g/l ^15^NH_4_Cl as the sole nitrogen source. Protein expression, lysis, and loading to Glutathione Sepharose™ column were performed as described above. The GST-tag was removed through on-column thrombin digestion by loading excess thrombin (SERVA) in wash buffer followed by overnight digestion at 4°C. The cleaved PHD moiety with a six-residue N-terminal extension (Gly–Ser–His–Met–Ala–Met–Ala) remaining from the thrombin cleavage site was collected from the flow-through and further purified in a Mono Q™ 10/100 GL as before and subsequently loaded to a Superdex-75 HiLoad 16/600 gel filtration column in NMR buffer (20 mM KPi, pH 7.0, 150 mM KCl, 10 μM ZnCl_2_) (Supplementary Fig. S1).

### H3 peptide synthesis

Peptides were synthesized at 25 μmol scale using standard Fmoc chemistry on tentagel S RAM resin (Rapp Polymere, Germany), automated on either a SyroII (Biotage, Sweden) or Chorus (Gyros Protein Technologies, USA) synthesizer. Deprotection was by 20% piperidine with 0.1 M ethyl cyanohydroxyiminoacetate (oxyma) as additive at 80°C for 90 s, followed by three washes with *N*,*N*-dimethylformamide (DMF). Coupling was with 5 equivalents (equiv.) of amino acid, 5 equiv. of oxyma, and 10 equiv. of *N*,*N*′-diisopropylcarbodiimide at 55°C for 15 min followed by capping with 2 M each acetic anhydride and pyridine in DMF for 5 min before again washing three times with DMF. Methylated lysine building blocks were incorporated as their Fmoc-protected building blocks. Serotonin was incorporated by peptide synthesis using an Fmoc-Glu(OAll)-OH building block, selectively deprotected by treating for 6 h with 3equiv. Pd(PPh_3_)_4_ in chloroform/acetic acid/*N*-methyl morpholine (volume ratio 37:2:1) under nitrogen flow throughout the reaction, before washing three times each with chloroform and DMF. Serotonin coupling was subsequently carried out by addition of 2 equiv. serotonin, 4 equiv. each benzotriazol-1-yloxytripyrrolidinophosphonium hexafluorophosphate (PyBOP) and 1-hydroxy-7-azabenzotriazole (HOAt), and 8 equiv. *N*,*N*-diisopropylethylamine (DIPEA) in DMF and allowing to react overnight, before washing three times with DMF. Peptide cleavage and global deprotection were carried out by treatment of the resin with a cleavage cocktail of TFA/water/1,2-ethanedithiol/triisopropylsilane (90:5:2.5:2.5) at room temperature for 2.5 h (*Note*: cleavage without thiol scavenger led to exclusively alkylated side product, reacting at the electron-rich serotonin ring), before precipitation of the product by addition to cold diethyl ether. The resulting solid was washed thrice with cold diethyl ether, then dried and purified by preparative high-performance liquid chromatography (HPLC) using a gradient of acetonitrile in water with 0.1% TFA modifier on a Gemini column 10uc18, 110A (10 μm, 21 mm × 250 mm) at a flow rate of 12.5 ml/min. Peptide purity was analysed by UV–HPLC using a 40-min gradient from 100% buffer A (95% water, 5% acetonitrile + 0.1% FA) to 70% buffer B (5% water, 95% acetonitrile + 0.1% FA) with a Silicycle C18 column (Dr Maisch, Germany) (150 mm × 4.6 mm, 5 μm), and peptide identity verified by electrospray ionization-mass spectrometry (Supplementary Fig. S2). Integrity of the (non-biotinylated) H3 peptides and presence of H3Q5ser were also confirmed by NMR (Supplementary Fig. S3).

### Peptide pull-down - SDS–PAGE

Biotinylated H3 (1–13) peptides immobilized on Dynabeads™ M-280 Streptavidin (Invitrogen) were incubated with bacterial crude lysate expressing GST-Taf3-PHD in binding buffer [50 mM Tris–HCl, pH 8.0, 150 mM NaCl, 0.5% NP-40, 10 μM ZnCl_2_, 0.5 mM PMSF, 0.5 mM DTT, 1× cOmplete™ Protease Inhibitor Cocktail (Roche)] for 2 h at 4°C while rotating. Plain streptavidin beads were used as control. Following extensive washing in binding buffer, bound proteins were eluted in Laemmli buffer, resolved by sodium dodecyl sulphate–polyacrylamide gel electrophoresis (SDS–PAGE), and stained with Bio-Safe Coomassie G-250 (Bio-Rad). The gel was imaged with a ChemiDoc™ Touch imaging system (Bio-Rad), and the resulting images were processed and analysed with the Image Lab 6.1 software (Bio-Rad) (Supplementary Fig. S4).

### Peptide pull-down - mass spectrometry

**Figure 1.**
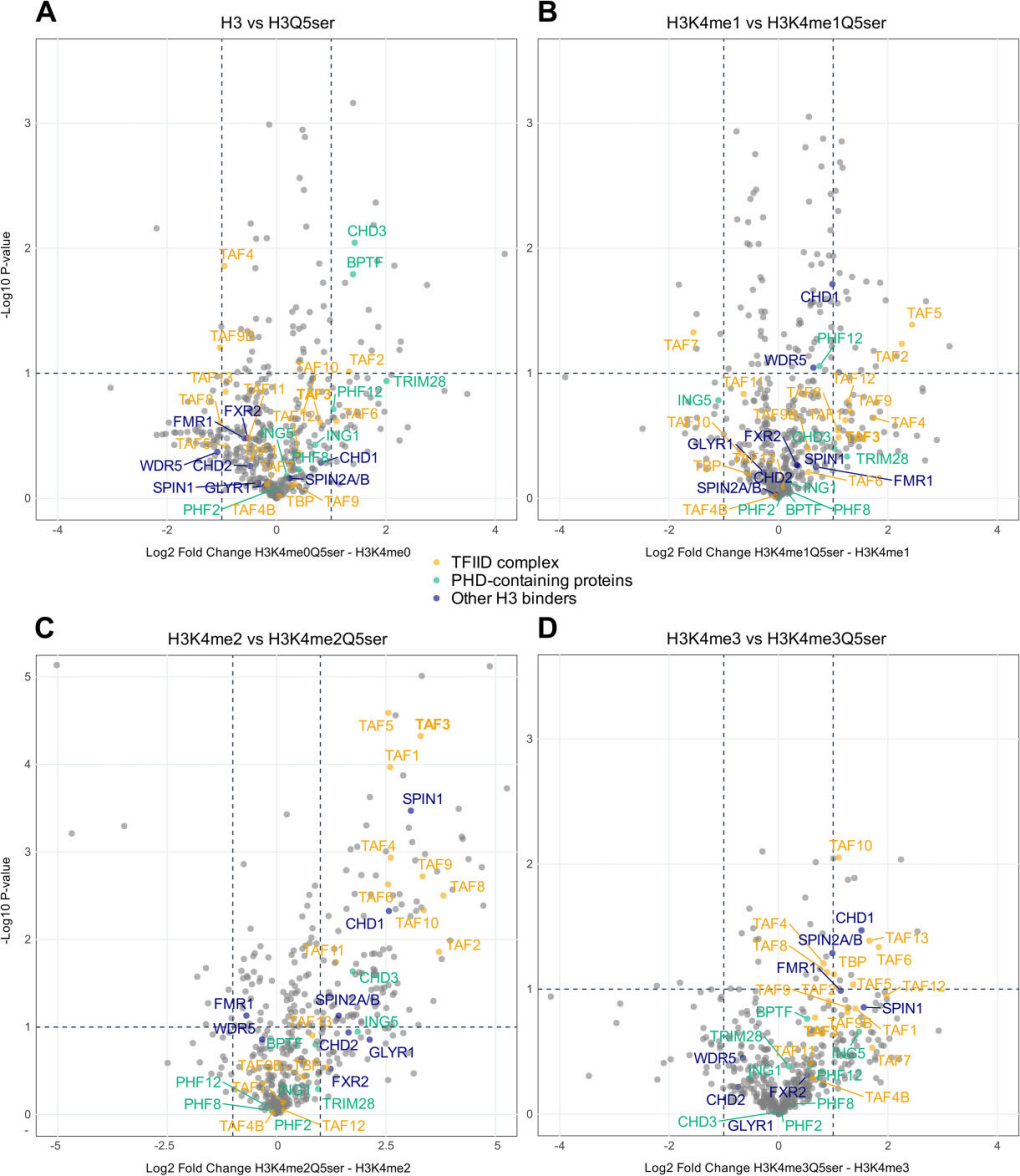
H3Q5 serotonylation enhances TFIID binding in all H3K4 methylation states. Quantitative mass spectrometry (qMS) confirms that TFIID has a preference towards H3Q5ser peptides in (**A**) H3K4me0, (**B**) H3K4me1, (**C**) H3K4me2, and (**D**) H3K4me3. False discovery rate cut-off is 0.1. Colour coding of identified proteins is indicated in the centre of the figure.

Nuclear extracts of HeLa cells were prepared using a modified version of the Dignam procedure [43]. Cytosolic and nuclear separation was validated by immunoblot using the following antibodies: for TBP the 20C7 in house mouse monoclonal and for Tubulin the DM1A monoclonal from Calbiochem (CP06) (Supplementary Fig. S5A). Biotinylated H3 (1–13) peptides immobilized on Dynabeads™ M-280 Streptavidin (Invitrogen) were incubated on a rotating wheel with 1 mg of HeLa nuclear extract overnight at 4°C in binding buffer [20 mM HEPES–KOH, pH 7.9, 300 mM NaCl, 20% glycerol, 2 mM MgCl_2_, 0.2 mM ethylenediaminetetraacetic acid, 0.1% NP-40, 0.5 mM DTT, and 1× cOmplete™ Protease Inhibitor Cocktail (Roche)]. The beads were then washed two times with binding buffer containing 0.5% NP-40, two times with phosphate buffered saline (PBS) containing 0.5% NP-40, and two times with plain PBS. On-bead digestion of bound proteins was performed overnight at 22°C with 0.1 μg/ml of trypsin in elution buffer (100 mM Tris–HCl, pH 7.5, 2 M urea, 10 mM DTT). The eluted peptides were then bound to C18 stage tips (Thermo Fisher), washed three times in 0.1% trifluoroacetic acid (TFA), eluted from the C18 stage tips in 65% acetonitrile and dried prior to resuspension in 10% formic acid. A third of the final elution was analysed as described in [44, 45] with minor changes. For nanoflow-HPLC–MS/MS samples were analysed on an Orbitrap Fusion Lumos mass spectrometer coupled to an Easy nano-LC 1200 HPLC (Thermo Fisher Scientific). Peptide separation was performed with a C18 separation column of 25 cm length (75 μm i.d., 2 μm particle size, 100 Ǻ pore size) with a gradient of two buffers with increasing organic proportion (buffer A: 0.1% formic acid; buffer B: 0.1% formic acid in 80% acetonitrile). The flow rate was 300 nl/min. For tandem MS, a TOP-10 method was used. Each MS scan was followed by a maximum of 10 MS/MS scans in the data-dependent mode. Data analysis was performed using MaxQuant (version 1.6.17.0) and Perseus (version 1.6.0.7) software (Fig. 1 and Supplementary Fig. S5B). All mass spectrometry data have been deposited to the ProteomeXchange Consortium via the PRIDE partner repository under the dataset identifier PXD062109.

### NMR

#### NMR samples and data collection

All data were recorded at 298 K on a Bruker Avance III HD 900 MHz spectrometer equipped with a cryoprobe, unless noted otherwise. Sample for assignment verification contained 0.3 mM ^13^C- and ^15^N-labelled PHD finger of Taf3 (residues 857–924) in NMR buffer (20 mM KPi, pH 7.0, 150 mM KCl, 10 μM ZnCl_2_, 0.01% NaN_3_, 10% D_2_O) in a 5-mm NMR tube. Samples for peptide interaction studies contained 0.19 mM ^13^C- and ^15^N-labelled PHD in NMR buffer in 3-mm NMR tubes. Lyophilized peptides were dissolved in NMR buffer including 1 mM sodium 3-(trimethylsilyl)tetradeuteriopropanionate (TSP) into 20 mM stock solutions. Upon dilution to 1 mM, all peptide solutions were at pH 7.0 in NMR buffer. Peptide concentrations were determined by comparing the integral of the Thr methyl signal (from T3, T6, and T11, total nine protons) to that of TSP using a quantitative 1D experiment (recycle delay 60 s) recorded at a Bruker Avance III 600 MHz spectrometer. Saturated complexes with H3K4me1/H3K4me2/H3K4me3 peptides with and without H3Q5ser were made by adding peptide to a 0.7 mM final concentration (3.6 equiv.). With expected dissociation constants (*K*_D_) of >0.2 mM, 1.7 μM, and 0.3 μM [32, 35] for H3K4me1, H3K4me2, and H3K4me3 peptides, respectively, the di- and trimethylated complexes are expected to be fully saturated, and the H3K4me1 complex should be saturated ∼70%. Additional samples were prepared using 30%, 50%, 80%, and 160% equiv. of peptide. All spectra were recorded and processed using Bruker Topspin and analysed using NMR-FAM Sparky [46] and Poky [47].

Samples for NOESY data collection contained 0.88 mM ^13^C- and ^15^N-labelled Taf3-PHD finger with 0.88 mM H3K4me3Q5ser peptide in low-salt NMR buffer (20 mM KPi, pH 7.0, 4 mM KCl, 10 μM ZnCl_2_, 0.01% NaN_3_, 10% D_2_O) in a 5-mm NMR tube. For structure determination, 2D ^13^C,^15^N F2-filtered NOESY experiments [48] were recorded at 293 K with 100, 150, and 200 ms mixing time and with and without ^13^C,^15^N decoupling during acquisition (for 150 ms mixing time only). In addition, a 2D F2-filtered TOCSY with 60 ms DISPI2 mixing time and 3D ^13^C-edited NOESY (optimized for either aliphatic or aromatic resonances) and ^15^N-edited NOESY–HSQC experiments with 100 ms mixing time were collected.

#### NMR chemical shift assignments

Backbone chemical shift assignments of free Taf3-PHD were verified using the deposited assignments (BMRB-id 15670) and a 3D HNCACB triple resonance experiment. Backbone assignment for the other complexes was transferred from the H3K4me3 complex making use of the titration data. Chemical shift assignments of the free peptides were based on 2D ^1^H–^1^H TOCSY and ^1^H–^13^C HSQC experiments. The ^1^H 1D spectrum with assignment and atom nomenclature of H3K4me3Q5ser is reported in Supplementary Fig. S3C. Side-chain chemical shift assignments of Taf3-PHD in the H3K4me3Q5ser complex were transferred from the non-serotonylated complex (BMRB ID 15671) and verified using 3D ^15^N- and ^13^C-edited NOESY spectra where necessary. Few residues showed significant chemical shift perturbations (CSPs) for their side-chain ^13^C and ^1^H chemical shifts: S880, P881, and W894. P881 is *in trans* conformation for both H3K4me3 and H3K4me3Q5ser complexes, as based on Cβ–Cγ chemical shift difference. Overall assignment completeness of Taf3-PHD was 97.5% for residues around the peptide binding site (N865–P912). The resonances of the H3K4me3Q5ser peptide in the bound state were completely assigned, with exception of R2, R8 Hη protons, the serotonin hydroxyl proton Q5 HSZ3, R8 Hε, and K9 Hα, using ^13^C-/^15^N-filtered TOCSY and NOESY spectra.

#### NMR titration data analysis

Peak intensities in spectra of free Taf3-PHD in complex with 3.6 equiv. of peptide were normalized by setting the intensity of C-terminal residue H924 to 1 in all spectra to account for small concentration differences across samples. For each H3K4 methylation state, the normalized intensities for the non-serotonylated complex were compared to those for the serotonylated complex. For the CSP, the high similarity of the chemical shift data for the different K4 methylation states was exploited to derive a common Qser effect. First, the average chemical shift for the non-serotonylated complexes (H3K4me1/H3K4me2/H3K4me3) and the average chemical shift for the complexes including H3Q5ser were calculated. Subsequently, the CSPs between these were derived using a weighting factor of 6.5 for the ^15^N chemical shift differences (expressed in ppm). Residues with serotonin-specific CSPs (nine in total) were defined as those with CSPs higher than the average CSP + one standard deviation, as based on the 50% smallest CSPs.

#### Determination of binding off-rate

Binding affinities and off-rates (*k*_off_) were estimated by fitting the line shapes of E862, G879, Y892, C896, A901, and C911 in spectra in the presence of 0%, 50%, and 150% peptide using the software TITAN [49] in MATLAB (v2016b, The Mathworks) (Supplementary Fig. S7). For the H3K4me2 complexes, the resonance of G879 was excluded due to overlap and that of K875 was included. Spectra were processed using NMRPipe [50] using exponential line broadening of 10/15 Hz in the direct/indirect dimension of the free induction decay before Fourier transform. Data were fit against a 1:1 binding model that also optimizes the ligand concentration to compensate for concentration errors. Fitting assumed an HSQC experiment using 1.2 ms for the first *zz*-filter delays and an additional 0.6 ms evolution at the start and end of t1 that accommodated the echo/anti-echo gradient. The average *k*_off_ value and standard error from a bootstrap analysis based on 100 replicas are reported. The 95% confidence limits were estimated using a grid search fixing *k*_off_ allowing for an ∼3% increase in the final residuals as based on the *F*-statistic given the degrees of freedom. For each *k*_off_ value, the *K*_D_ for the H3K4me3 peptide was restrained to fall within 100 and 400 nM to enforce compatibility to previous reported affinity [23, 32, 35] to avoid unrealistically high fitted *K*_D_ values at higher *k*_off_ values. Of note, the quality of the fit at the confidence limit edges is visibly worse than the best fit, particularly for the high *k*_off_ side. For the H3K4me3Q5ser peptide, no restraint on *K*_D_ was necessary. For the H3K4me2 peptides, the *K*_D_ was restrained to 5 μM maximum. Without these upper bounds for *K*_D_, slightly wider confidence limits for *k*_off_ are found.

#### Structure calculation

The structure of the Taf3-PHD/H3K4me3Q5ser complex was calculated using a hybrid approach: distance restraints obtained from 2D F2-filtered NOESY and 3D ^13^C- and ^15^N-edited spectra for peptide–peptide, peptide–protein, and interface protein–protein contacts were imposed on the structure of the H3K4me3–TAF-PHD complex (PDB ID 2K17), as outlined below. NOEs in the F2-filtered NOESY spectra with 150 ms mixing time were compared to the spectrum obtained with ^13^C/^15^N decoupling to verify that these were either intra-peptide or protein–peptide NOEs and not from a ^13^C-bound proton that may have leaked through the filter. Intermolecular NOEs were assigned using the previous data on the H3K4me3 complex. New intermolecular NOEs for H3Q5ser were verified in the 3D NOESY spectra where possible. Additionally, intra-protein NOEs were assigned for the residues in and around the peptide binding site from either NOEs or CSPs (residues 865, 866, 868, 869, 874, 876–883, 893–895, 897–903, 908, 909, and 916). Assigned NOEs with signal-to-noise ratios >8 in the F2-filtered 2D NOESY, 3D aliphatic and aromatic optimized 3D ^13^C-edited NOESY, and the 3D ^15^N-edited NOESY were converted to unambiguous distance restraints. Intensities of reciprocal NOEs were averaged and converted to a single distance restraint. NOEs involving diastereotopic methylene protons were made ambiguous to either proton, taking the highest intensity for distance calibration. The NOE intensities were calibrated such that converted distances were in the 2.1–7.1 Å range. See Supplementary Table S1 for statistics on the distance restraints.

The structure of the Taf3-PHD/H3K4me3Q5ser complex was calculated using HADDOCK 2.4 [51, 52], taking the structure of the Taf3-PHD/H3K4me3 complex (PDB ID 2K17) as the starting conformation for protein and peptide. The topology definitions were extended to include serotonylated glutamine (three-letter code QSR; see Supplementary Table S2) based on the parameters of tryptophan and serotonin taken from the Automated Topology Builder website (http://compbio.biosci.uq.edu.au/atb/) [53]. Residues 6–12 of the peptide were defined as fully flexible. The ensemble of input structures was used with cross-docking. In the rigid body stage, 1200 solutions were calculated, after which the best 400 scoring solutions were subjected to semi-flexible refinement followed by refinement of the best 200 solutions in explicit water.

Initial calculations using all intermolecular distance restraints (113) resulted in two distinct conformations for H3Q5ser: one in which the serotonin group is stacked in an edge–face (T-shaped) arrangement on P881 and W894 (9/20 structures) and one in which the serotonin group is parallel to and roughly on top of W894 (11/20 structures) (see Supplementary Fig. S8C). In all nine T-stack or edge–face structures, NOEs Q5 HSD1 –A901 HA and Q5 HSE1 –A901 HA violated by >2.5 Å. Comparison of NOESY spectra recorded at different mixing times revealed that these NOEs are comparatively stronger at longer mixing times than others, suggesting that the NOEs may be caused by spin diffusion (Supplementary Fig. S8B and D). One-by-one removal of these NOEs in order of violation magnitude resulted in an ensemble with H3Q5ser exclusively in an edge–face conformation (Fig. 4 and Supplementary Fig. S9).

The final structure calculation was performed after exclusion of two NOEs (Q5 HSD1 –A902 QB; Q5 HSZ2 –A901 QB) that lacked structural support, resulting in a final ensemble without any consistent violations (four violating NOEs in the top 40 structures, with each violating NOE occurring in one or two structures). The final 20 best-scoring solutions without NOE violations >0.5 Å (out of the top 24 structures according to HADDOCK score) were taken as the final ensemble of solutions and analysed, including reporting of violations statistics and energy interaction scores per residues. Structural superposition and calculation of root mean square deviations (RMSDs) were performed using ProFit V3.3 (A.C.R. Martin and , C.T. Porter, http://www.bioinf.org.uk/software/profit/). The most representative structure according to HADDOCK was taken to represent the ensemble and used for illustrations.

### Isothermal titration calorimetry

ITC experiments were performed at 20°C with a MicroCal VP-ITC microcalorimeter (Malvern) by sequential injection of 10 μl at 300 μM histone peptide during 20 s into the sample cell containing 30 or 15 μM of GST-Taf3 (wild type or mutant) or GST-BPTF, GST-PHF2, and GST-PHF8 PHDs in ITC buffer (100 mM NaCl, 20 mM Tris–HCl, pH 7.5). A total of 29 injections were done with a 240 s spacing in between. ITC data were subsequently analysed and fitted with the one-site binding model in the MicroCal ITC Origin Analysis software 7.0 (Table 1, Figs 3B and 5A, Supplementary Table S3, and Supplementary Fig. S10). Final figures were merged and plotted using the OriginPro software (version 10.15 2024b).

**Table 1.** Affinities, ratios, and significance of the ITC results obtained with GST-tagged PHDs as shown in Figs 3B and 5A

Protein	Peptide	*K*_D_ (μM)	*K*_D_ ratio (H3K4me3/ H3K4me3Q5ser)	*Z*-score ratio (H3K4me3/ H3K4me3Q5ser)	Significance (99% confidence)	Affintiy change upon H3Q5ser
TAF3 WT	H3K4me3	0.260 ± 0.016	2.66 ± 0.28	5.94	True	Increased
	H3K4me3Q5ser	0.098 ± 0.008				
TAF3 A902L	H3K4me3	0.166 ± 0.017	3.25 ± 0.40	5.63	True	Increased
	H3K4me3Q5ser	0.051 ± 0.003				
TAF3 S880A	H3K4me3	0.207 ± 0.034	3.20 ± 0.59	3.73	True	Increased
	H3K4me3Q5ser	0.065 ± 0.005				
TAF3 P881E	H3K4me3	0.148 ± 0.004	1.09 ± 0.04	1.97	False	No change
	H3K4me3Q5ser	0.136 ± 0.004				
TAF3 P881F	H3K4me3	0.130 ± 0.004	1.05 ± 0.06	0.82	False	No change
	H3K4me3Q5ser	0.124 ± 0.007				
TAF3 W894F	H3K4me3	0.126 ± 0.012	1.24 ± 0.12	2.04	False	No change
	H3K4me3Q5ser	0.102 ± 0.001				
TAF3 W894I	H3K4me3	0.279 ± 0.017	2.64 ± 0.25	6.45	True	Increased
	H3K4me3Q5ser	0.106 ± 0.008				
TAF3 W894L	H3K4me3	0.595 ± 0.035	1.84 ± 0.15	5.66	True	Increased
	H3K4me3Q5ser	0.324 ± 0.018				
TAF3 P881E W894F	H3K4me3	0.123 ± 0.010	1.11 ± 0.16	0.65	False	No change
	H3K4me3Q5ser	0.111 ± 0.014				
BPTF	H3K4me3	1.297 ± 0.049	1.05 ± 0.06	0.85	False	No change
	H3K4me3Q5ser	1.236 ± 0.050				
PHF2	H3K4me3	0.286 ± 0.023	1.42 ± 0.19	2.21	False	No change
	H3K4me3Q5ser	0.201 ± 0.022				
PHF8	H3K4me3	0.418 ± 0.020	1.52 ± 0.13	4.05	True	Increased
	H3K4me3Q5ser	0.275 ± 0.019				

Representative experiments are shown for each GST-fusion protein. Curve fitting analysis was performed following the one-set-of-sites model in the MicroCal-ITC Origin 7 software. Uncertainties reflect standard errors obtained from the curve fitting process using a non-linear least squares model on individual datasets.

### Multiple sequence alignment

To study conservation of the PHD domain in the human proteome, full-length sequences of 105 human PHD-containing proteins were downloaded from the UniProt database and compiled into a curated FASTA file. The consensus hidden Markov model (HMM) for the PHD superfamily domain ‘pfam00628’ was obtained from the InterPro database. To identify the positions of PHD domains within the protein sequences, the hmmscan tool from the HMMER3 package (version 3.4; http://hmmer.org) was used with default parameters. The search was performed using the PHD domain profile (PF00628.hmm) against the curated FASTA file of full-length proteins. Domain hits with independent *E*-values below 0.01 were retained. This led to the identification of a total of 136 PHD fingers within the full-length protein sequences so that the corresponding sequences, extending five amino acids upstream and downstream of the identified domains, could be extracted. The list was reduced to 108 after excluding PZP domains and other domains with incomplete Cys4–His–Cys3 PHD motif. These sequences were aligned using the ClustalW tool (version 2.1) in the galaxy platform [54] followed by manual curation (Fig. 5B and Supplementary Fig. S11).

Similarly, the available full-length TAF3 protein sequences from a selection of organisms were downloaded from UniProt. The positions of PHD domains within the protein sequences were identified using the hmmscan tool from the HMMER3 package (version 3.4; http://hmmer.org) with default parameters. Finally, these sequences were aligned using the ClustalW tool (version 2.1) in the galaxy platform [54] (Supplementary Fig. S12).

## Results

### TFIID and TAF3-PHD engage with H3Q5ser peptides in all H3K4 methylation states

TFIID, through the PHD finger of TAF3, shows a clear preference for H3K4me3 but can still engage with di-, mono-, and unmethylated H3K4, albeit with decreasing affinity [23, 32, 35]. As recent *in vitro* data indicate that H3Q5ser potentiates TAF3-PHD binding in all methylation states [23], we sought to validate the effect of H3Q5 serotonylation on TFIID binding to unmethylated H3, H3K4me1, H3K4me2, and H3K4me3 in cellular extracts. Histone peptide pull-downs followed by qMS were performed using HeLa cell nuclear extract. Comparing enrichment of singly or doubly modified H3 peptides showed that TFIID favours H3Q5ser peptides over the non-serotonylated counterparts, in all H3K4 methylation states (Fig. 1A–D). TFIID enrichment was most pronounced for the H3K4me2Q5ser peptide. The PHD-containing protein BPTF displayed enhanced binding to H3Q5ser compared to the unmodified H3 peptide, but this preference was lost with the mono-, di-, and trimethylated H3K4 peptides (Fig. 1A–D). Similarly, CHD3 displayed a preference for H3Q5-serotonylated H3K4me0 and H3K4me2 peptides but not for the H3K4me1 and H3K4me3 peptides. The other PHD-containing proteins detected in this experiment did not display any clear preference or aversion towards H3Q5ser. Additional H3 binding proteins were preferentially enriched in the serotonylated di- and trimethylated samples. This is the case for CHD1 and SPIN1, the H3 binding domains of which have been reported to have a 2.2- and 1.5-fold stronger binding affinity for H3K4me3Q5ser compared to H3K4me3, respectively [23] (Fig. 1C and D). In these samples, SPIN2A/B displayed a similar preference for serotonylated H3Q5 (Fig. 1C and D).

To confirm these observations, the binding of recombinant Taf3-PHD finger to immobilized H3 histone tail peptides was assessed by pull-down experiments. Again, the set of peptides tested spanned all possible H3K4 methylation and H3Q5 serotonylation states. As expected, the Taf3-PHD bound very weakly to the non-methylated peptides and binding notably increased with H3K4 methylation. The strongest stimulation by H3Q5ser was observed with H3K4me2 (Supplementary Fig. S4).

These findings are consistent with ITC measurements of recombinant TAF3-PHD, SPIN1-Ssty, and CHD1-chromodomain [23]. In addition, these results validate that both TFIID and recombinant Taf3-PHD display a preference towards H3Q5ser, which is not shared with other nuclear PHD proteins.

### H3Q5ser creates a novel binding surface on Taf3-PHD

While a crystal structure of TAF3-PHD with H3K4me3Q5ser is available (PDB: 5XMY), the serotonin group is not resolved. In previous work, we elucidated the 3D structure of the Taf3-PHD/H3K4me3 complex by solution NMR methods [32]. Therefore, we applied these methods to obtain structural insight into H3Q5ser binding by Taf3-PHD. Addition of mono-, di-, or trimethylated H3K4 peptides with or without H3Q5 serotonylation resulted in clear CSPs of Taf3-PHD resonances. Chemical shifts of free and H3K4me3-bound Taf3-PHD were nearly identical to those observed before [32], allowing straightforward transfer of the peak assignments.

**Figure 2.**
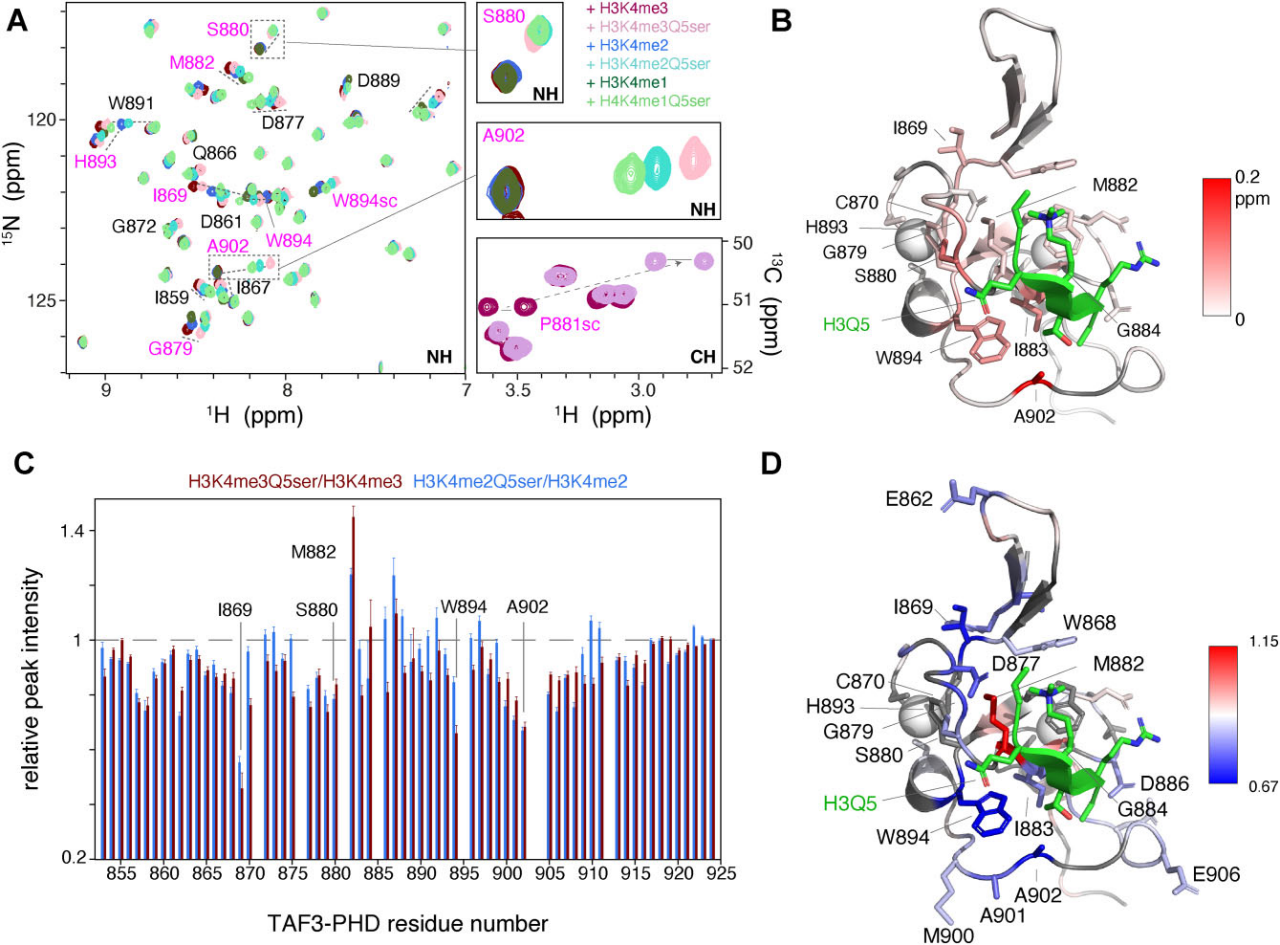
NMR analysis of Taf3-PHD finger bound to H3K4me peptides with and without H3Q5ser. (**A**) Overlay of 2D ^15^N-HSQC (left) and ^13^C-HSQC (proline Cδ–Hδ region, bottom right) spectra of Taf3-PHD with excess of indicated peptides. Resonances with significant CSPs are labelled and connected by dashed lines. Residues with H3Q5ser-dependent chemical shifts are indicated with magenta labels, with A902 and S880 highlighted and expanded in the boxes. (**B**) Structure of Taf3-PHD bound to H3K4me3 peptide (in green) (PDB 2K17) colour coded with CSPs between average chemical shift of the non-serotonylated complexes (H3K4me3/H4K4me2/H3K4me1) and the corresponding Q5 serotonylated complexes. (**C**) Normalized peak intensities of non-overlapping Taf3-PHD residues in complex with H3K4me3Q5ser and H3K4me2Q5ser peptides, relative to their non-serotonylated counterparts. (**D**) As the panel (B), but here colour coded according to the normalized peak intensity for the H3K4me3Q5ser complex, relative to the H3K4me3 complex. Selected residues are labelled. Colour coding indicated in the figure.

Comparison of the spectra of the different H3 peptide complexes showed that while for most resonances the chemical shifts of Taf3 residues are highly similar, a few displayed H3Q5ser-specific chemical shifts (Fig. 2A and Supplementary Fig. 6). Chemical shifts that are mostly sensitive to the H3K4 methylation status, e.g. the side-chain peaks of W868 and W891, are part of the trimethyl lysine cage (Supplementary Fig. S6). In contrast, others like A902 and S880 are highly sensitive to H3Q5 serotonylation (Fig. 2A insets). CSP analysis showed that the residues with serotonin-specific chemical shifts across all three H3K4 methylation states cluster on the Taf3-PHD surface around H3Q5 (Fig. 2B). In addition, comparison of Taf3-PHD side-chain chemical shifts between the H3K4me3 and H3K4me3Q5ser complex revealed pronounced CSPs for S880, P881, and W894 (Fig. 2A and Supplementary Fig. S6A). Interestingly, residues in the H3K4me binding cage and around the neighbouring zinc-coordination site also show serotonylation-dependent CSPs. The H3K4me binding cage ‘floor’ M882 has a higher relative intensity in the H3Q5ser complexes. The pronounced peak broadening of M882 in the non-serotonylated complexes is not observed for H3Q5ser complexes. This suggests that the presence of H3Q5ser rigidifies the binding pocket for methylated H3K4. On the other hand, S880, W894, and A902 and neighbouring residues have reduced peak intensities in the serotonylated complexes, suggesting additional line broadening due to conformational exchange.

Overall, these data suggest that the Taf3-PHD surface that includes S880, P881, W894, and the loop containing A902 forms an additional interaction surface for the serotonin group. Moreover, the presence of H3Q5ser could further stabilize the H3K4me3 aromatic cage.

### H3Q5 serotonylation increases complex life time and further stabilizes Taf3-PHD–H3K4me3 interaction

The peak positions and line shapes of NMR signals are very sensitive to populations of free and bound proteins and their dynamic interconversion in a protein-ligand binding equilibrium. By fitting the peak displacement and line shape of a protein resonance during a ligand titration, the binding affinity and kinetics can be fitted [55]. In particular, the line shapes of resonances around the binding midpoint are very sensitive to the binding off-rate *k*_off_, which is strongly correlated to the binding affinity. By fitting the spectra of free, fully bound, and ∼50% bound Taf3-PHD, the binding off-rates (*k*_off_) for the H3K4me2 and H3K4me3 complexes with and without serotonylation were determined.

**Figure 3.**
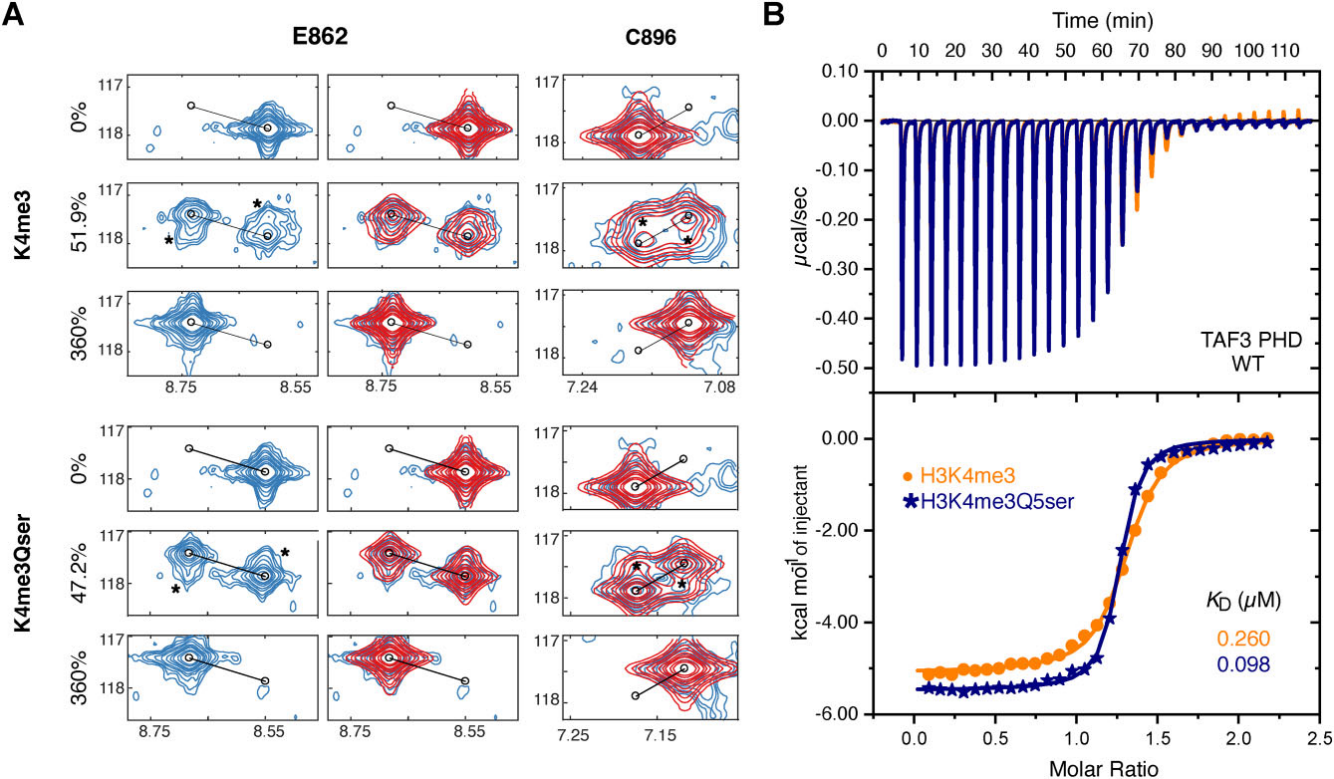
H3Q5ser stabilizes Taf3-PHD/H3K4me2 and Taf3-PHD/H3K4me3 complexes. (**A**) Experimental (blue) and fitted (red) spectra of free, ∼50% bound, and fully bound TAF3-PHD resonances E862 and C896 for H3K4me3/H3K4me3Q5ser complex. Transfer peaks indicated with *. Experimental spectrum of E862 is also shown separately for clarity. (**B**) ITC analysis of GST-tagged Taf3-PHD binding to H3K4me3 in yellow and H3K4me3Q5ser in navy blue. Top panel displays the raw heat change per injection of H3 peptide. Bottom panel represents the binding isotherm. The solid line represents the best fit to the one-set-of-sites model using MicroCal-ITC Origin 7 software.

Examination of the midpoint spectra shows clear transfer peaks between free and bound forms for the H3K4me3 peptide, indicating (slow) interconversion on the timescale of the experiment (Fig. 3A, peaks marked with asterisk). In contrast, transfer peaks are much reduced in intensity for the serotonylated H3 peptide complex, indicating slower interconversion between free and bound forms (Fig. 3A and Supplementary Fig. S7). Fitting of the line shapes in these spectra using TITAN [49] shows that *k*_off_ drops from 48.8 ± 1.0 s^−1^ (95% confidence limits 42–56 s^−1^) for H3K4me3 to 16.8 ± 0.5 s^−1^ (95% confidence limits 12–22 s^−1^) for the serotonylated H3K4me3 peptide (Fig. 3A and Supplementary Fig. 7A). For the H3K4me2 peptides, several resonances show strong line broadening around the midpoint for H3K4me2, but show transfer peaks for H3K4me2Qser, again indicating a reduction in *k*_off_ upon serotonylation. Line shape fits indicate that *k*_off_ drops from 207 ± 10 s^−1^ (95% confidence limits 160–250 s^−1^) to 71 ± 2s^−1^ (95% confidence limits 56–90 s^−1^) upon serotonylation of the H3K4me2 peptide (Supplementary Fig. 7B).

Assuming the binding on-rate is the same for all peptides, which is not unlikely given the minor chemical differences in the peptide, serotonylation increases the binding affinity of Taf3 ∼2.9-fold for both tri- and dimethylated H3K4 peptides by increasing the lifetime of the complex. Subsequent ITC measurements showed an ∼2.6-fold increase in binding affinity, in very good agreement with the NMR results. The affinities of Taf3-PHD were determined to be 0.098 ± 0.008 and 0.260 ± 0.016 μM, for H3K4me3Q5ser or H3K4me3 peptides, respectively (Fig. 3B and Table 1). Analysis of the fitted Δ*H* and Δ*S* values highlights that serotonylation increases both enthalpic and entropic contribution to the binding energy (Supplementary Fig. S10). At 293 K, the enthalpic stabilization contributes most to the increased binding affinity, indicative of formation of new stabilizing intermolecular interactions between H3Q5ser and the Taf3-PHD surface.

### Structure of Taf3-PHD in complex with the dual histone modification H3K4me3Q5ser

**Figure 4.**
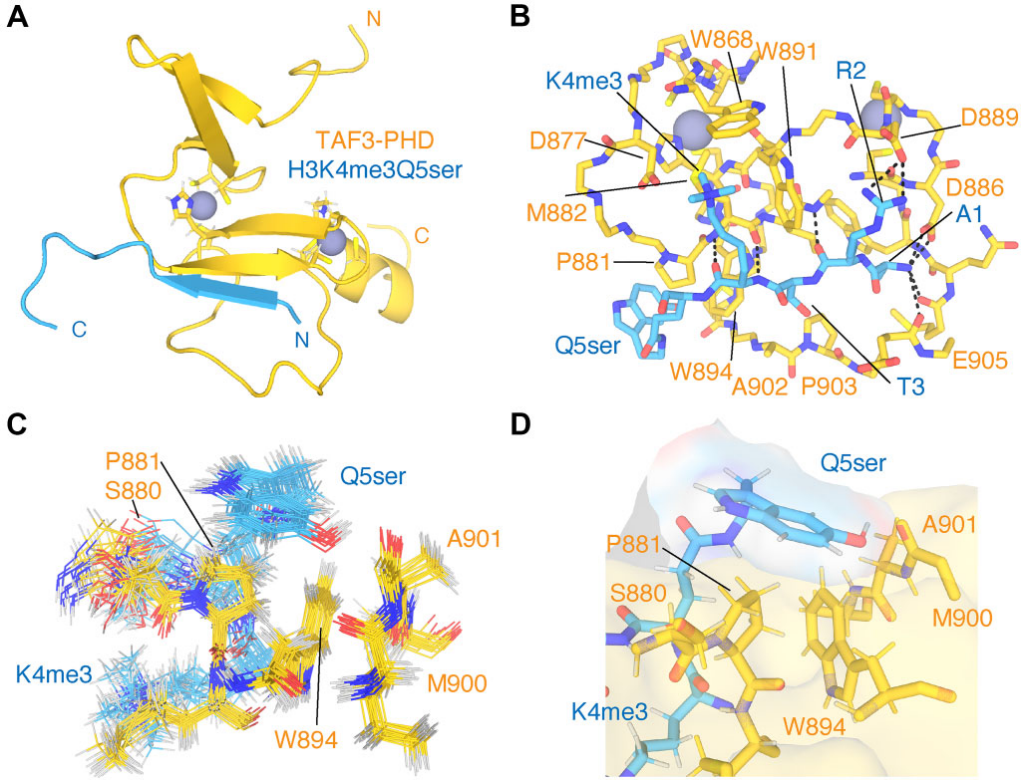
Solution NMR structure of the Taf3-PHD/H3K4me3Q5ser complex. (**A**) Most representative structure of the complex in cartoon depiction. Coordinated zinc atoms shown as spheres, with coordinating residues in sticks. (**B**) Close-up on the binding interface with intermolecular hydrogen bonds conserved across the ensemble shown as black dashed lines. (**C**) Superposition of the ensemble of 20 solution structures, zoomed in on the Q5ser binding site. (**D**) Close-up on the Q5ser binding site showing the molecular surfaces of Taf3-PHD and H3K4me3Q5ser to illustrate the stacking of H3Q5ser on P881 and W894.

We next collected a set of NOESY experiments resulting in 109 intermolecular distance restraints of which 35 originated from H3Q5ser. These include clear and reciprocal NOEs between Taf3-PHD P881 and the serotonin group (Supplementary Fig. S8A). Based on these and on peptide–peptide and protein–protein restraints, the structure of the Taf3-PHD bound to H3K4me3Q5ser was calculated in a hybrid approach starting from the TAF-PHD/H3K4me3 structure (see the ‘Materials and methods’ section and Supplementary Table S1). The final ensemble of structures contains no violations and is very similar to the structure of the H3K4me3 complex: 1.14 Å heavy backbone atom RMSD for peptide residues 1–4 and 0.98 Å for Taf3-PHD (Supplementary Table S1). Overall, the structure is well defined, including the H3Q5ser binding surface (Fig. 4 and Supplementary Fig. S9). The H3 tail peptide adopts a β-strand conformation and is bound in a groove formed by the β4-strand and the αC helix, extending the central β-sheet of the Taf3-PHD (Fig. 4A and B). The interactions of H3 tail residues 1–4 with Taf3-PHD are as in the H3K4me3 complex: the N-terminus is buried and forms multiple hydrogen bonds with Taf3-PHD side-chain carboxyl and backbone carbonyl groups; the R2 guanidium group is hydrogen bonded to D889 or E905; the methyl group of the H3T3 side chain makes contacts with the hydrophobic residues I883 in the binding groove; and H3K4me3 is bound in the aromatic cage (D877, M882, W868, and W891) (Fig. 4B).

The H3Q5ser residue forms a cap on the end of the binding groove through close contacts of the serotonin group with Taf3-PHD (Fig. 4C and D). The aromatic serotonin ring stacks in an edge–face arrangement on top of the side chains of P881 and W894. The serotonin rings sit on top of the proline ring in a conformation that is typical of supporting favourable CH–π aromatic–proline interactions [7]. Additionally, the W894 side chain contributes to the binding surface for the serotonin ring. While their aromatic rings do not directly overlap, precluding CH–π interactions, the W894 CE3/HE3 group contacts the serotonin hydroxyl group, indicative of a CH–O interaction [56]. Only few structures in the ensemble also show an intermolecular hydrogen bond from the serotonin hydroxyl group, suggesting that the aromatic ring is the dominant force of the serotonin-specific interaction. This is largely in agreement with ITC data testing several serotonin derivatives for binding to TAF3-PHD, which indicated that the electron orbitals of H3Q5ser determine the increase in binding affinity [23].

In conclusion, our Taf3-PHD/H3K4me3Q5ser structure supports an overall histone binding mode for the H3K4me3Q5ser as observed for the H3K4me3 peptide and, importantly, additional edge–face, foremost aromatic–proline, interactions are present between the H3Q5ser and the P881–W894 Taf3 amino acids.

### TAF3-PHD residues P881 and W894 are crucial for serotonin specificity

**Figure 5.**
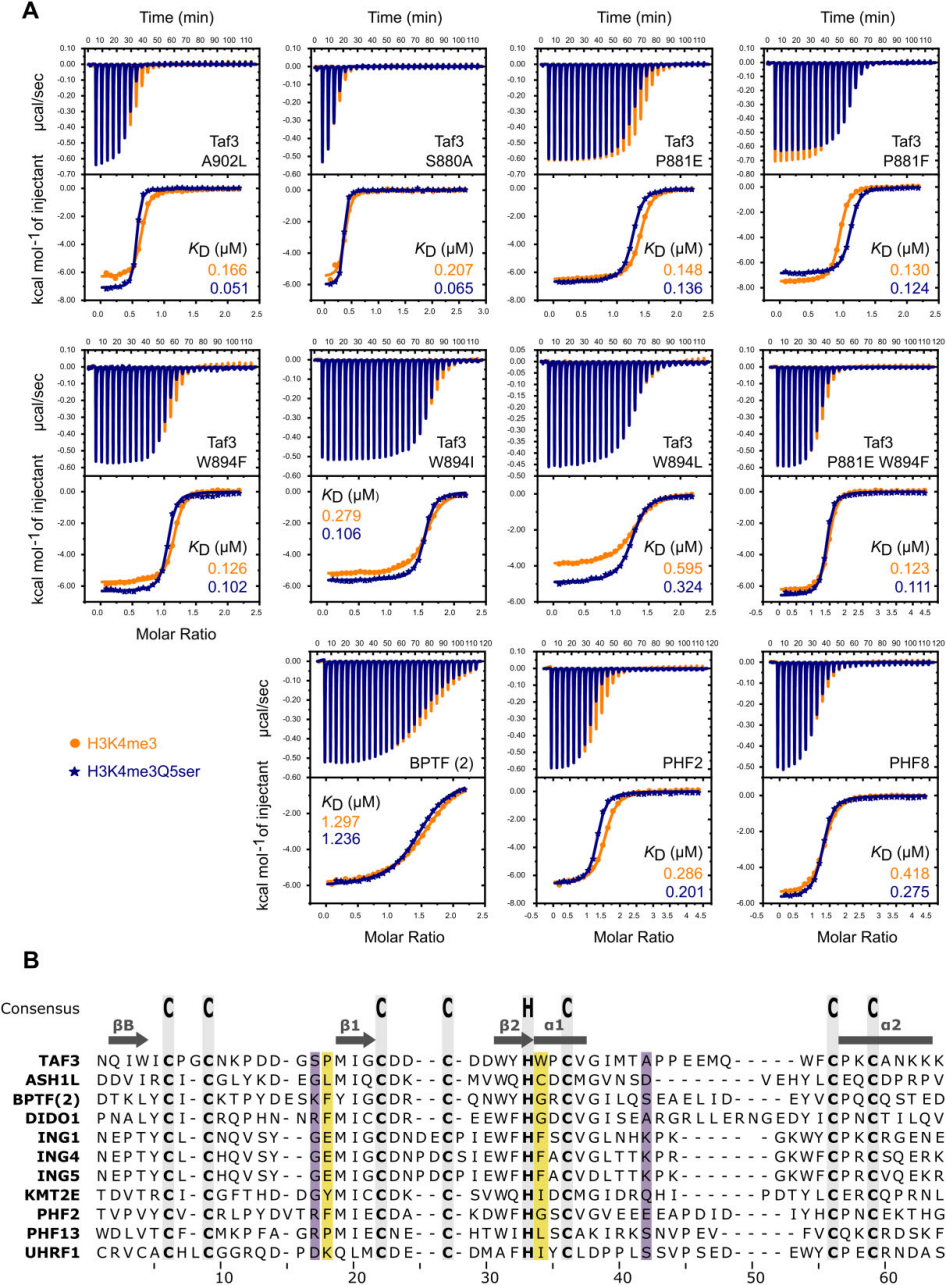
Taf3-PHD P881 and W894 form key interaction surface with the serotonin group. (**A**) ITC analysis of GST-tagged Taf3-PHD mutants, BPTF-PHD [2], PHF2-PHD, or PHF8-PHD with the peptides H3K4me3 (in yellow) and H3K4me3Q5ser (in blue). Top panels display the raw heat change per injection of H3 peptide. Bottom panels represent the binding isotherm. The solid line represents the best fit to the one-set-of-sites model using MicroCal-ITC Origin 7 software. (**B**) Multiple sequence alignment (MSA) of human PHD fingers for which high-resolution structures in complex with H3K4me3 are available (ASH1L PDB: 8VLF; BPTF(2) PDB: 2FUU; DIDO1 PDB: 4L7X; ING1 PDB: 2QIC; ING4 PDB: 2PNX/2VNF; ING5 PDB: 3C6W; KMT2E PDB: 4L58; PHF2 PDB: 3KQI; PHF13 PDB: 3O7A; UHRF1 PDB: 3SOW). Consensus Cys4–His–Cys3 positions are highlighted in grey and indicated on top. Locations of the secondary structure elements of TAF3-PHD are indicated on top. S880 and A902 are highlighted in purple, while P881 and W894 are highlighted in yellow.

With H3Q5 serotonylation shown to improve binding and extend the H3 peptide interaction surface on Taf3-PHD, the next step was to investigate the binding mode in greater detail by determining the affinity of Taf3-PHD mutants using ITC. The mutations targeted Taf3 residues with the largest serotonin-specific CSPs (S880, P881, W894, and A902) (Fig. 5A and Supplementary Fig. S6). Each residue was mutated to an alternative amino acid frequently found in other PHD fingers, as indicated by sequence alignments (Supplementary Fig. S11).

Taf3 mutants A902L as well as S880A preserved a three-fold increased affinity towards H3K4me3Q5ser compared to H3K4me3. However, mutations of P881 into either a glutamic acid or a phenylalanine disrupt the increased affinity towards H3K4me3Q5ser. Exchanging W894 with the less bulky phenylalanine abolished the preference for the H3Q5ser state. However, substitution of W894 to isoleucine or leucine retains a two- to three-fold increased binding to the serotonylated peptide, suggesting that stacking of serotonin ring on P881 is sufficient to support increase affinity. As expected by the single mutants and the NMR structure, the double mutant P881E–W894F lacks any preference towards H3K4me3Q5ser (Fig. 5A and Table 1). Thus, key interactions for H3Q5ser enhancement of Taf3-PHD binding to H3K4me3 include P881 and W894, but not S880 or A902.

To support these results and validate the lack of H3Q5ser specificity, NMR titration experiments of Taf3-PHD P881E–W894F with H3K4me3 or H3K4me3Q5ser peptides were performed. NMR analysis indicated that the mutant protein is less stable as indicated by several backbone amide resonances of residues at and around the mutation sites displaying low peak intensities or broadened beyond detection (Supplementary Fig. S13). Addition of H3K4me3 peptides with and without H3Q5 serotonylation results in clear CSPs that are similar to that observed for wild-type protein (Supplementary Fig. S13) indicating that the overall binding mode is maintained for the mutant. Strikingly, the best-fit *k*_off_ for both complexes are very similar in case of the double mutant: 29.6 ± 0.9 s^−1^ (22–40 s^−1^ 95% confidence limits) for non-serotonylated peptide versus 24.2 ± 0.9 s^−1^ (18–33 s^−1^ 95% confidence limits) for the H3K4me3Qser peptide (Supplementary Fig. S14). Noticeably, the *k*_off_ for the H3K4me3 peptide is reduced for the mutant compared to wild-type (29.6 s−1 versus 48.8 s^−1^), in line with the decreased dissociation constants obtained by ITC. As the P881E–W894F mutation may destabilize the protein, stabilization of the protein fold upon binding of the peptide can result in an enhanced affinity.

Determining the crucial role of the Taf3 P881 and W894 for H3Q5ser binding motivated us to determine the occurrence of these two residues among other PHD fingers. MSA using all the PHD-containing proteins found in the human proteome revealed that TAF3-PHD is the only PHD finger with the combination of proline and tryptophan in the determined positions (Fig. 5B and Supplementary Fig. S11).

Additionally, MSA of the PHD domains of TAF3 orthologues revealed a high degree of proline and tryptophan conservation in the positions corresponding to 881 and 894 across evolutionary distant species (Supplementary Fig. S12). This observation strengthens the importance of these two TAF3 residues for H3Q5ser recognition. It is important to note that serotonin as a signal molecule and potentially also as a histone modification appeared very early in evolution [15, 57]. In conclusion, we propose that a proline–tryptophan pair as present in the TAF3-PHD provides a conserved binding surface for the serotonin group attached to histone H3Q5.

## Discussion

Understanding the molecular determinants of histone H3Q5ser recognition by TFIID provides insight into the link between serotonylated chromatin and the pol II transcription machinery. In this study, we have determined the solution structure of Taf3-PHD in complex with the H3K4me3Q5ser-modified peptide. Supported by a range of qMS, ITC, and solution NMR data, we find that H3Q5 serotonylation enhances the affinity of the Taf3-PHD for the H3 tail about three-fold. The solution NMR structure revealed that H3Q5 serotonylation does not affect the position adopted by H3K4me3 in the aromatic cage, as H3K4me3Q5ser interacts with Taf3-PHD with very similar conformation as with H3K4me3. Still, H3Q5ser may indirectly contribute to increased affinity by rigidifying and/or stabilizing the H3K4me3 binding pocket. Importantly, the decisive, stabilizing interactions are formed by the serotonin moiety with P881, through proline–aromatic CH–π interactions [58–60], with additional support from van der Waals contacts and possibly CH–O interactions with W894 (Fig. 4). Mutational analysis of Taf3-PHD supports the solution NMR model for the H3Q5ser interaction. While mutation of P881 can completely abolish the preferential binding, not all W894 mutations lead to a complete loss of H3Q5ser preference (Fig. 5A). Together, this indicated that both P881 and W894 interact with H3Q5ser and that the proline/serotonin interaction has a higher contribution to the preference for H3Q5ser. In this light, its notable that the CSPs for P881 ring ^1^H and ^13^C chemical shifts were much larger than for W894 (Fig. 2 and Supplementary Fig. S6), consistent with closer distance to the aromatic serotonin rings seen in the structure and the thus larger expected ring current shifts.

Both the NMR analysis of complex off-rate and the ITC affinity data consistently show a three-fold preferential binding towards H3Q5ser in the H3K4 di- and trimethyl conditions. Consistent with the affinity values, semi-quantitative H3 peptide pull-down experiments indicated a mild preference towards H3Q5ser. Previous studies reported larger changes in affinity (between five- and nine-fold) [23]. These studies also reported a two- to five-fold increased binding to H3Q5ser by the PHD finger of ING2, the Tudor domain of SGF29, and the CW-Zf domain of ZCWPW1. None of these proteins were identified as interactors of H3 peptides in the qMS experiment (Fig. 1). The qMS and ITC data (Figs 1 and 5A, and Table 1) do not show an effect of H3Q5ser on BPTF binding, whereas a two-fold decrease was reported previously [23]. WDR5 was similarly neutral to serotonylation as indicated by qMS (Fig. 1) despite ITC determining a two-fold enhanced binding in both H3K4me0 and H3K4me3 [61]. Possibly, the lack of WDR5 preference for H3Q5ser in the qMS experiment (Fig. 1) is related to binding of MLL peptides to the WDR5 cavity also used by H3Q5ser [61–63]. On the other hand, the qMS experiment confirms the preference of CHD1 and SPIN1 for serotonylated chromatin (Fig. 1C and D) [23] and suggests that SPIN2A/B binding to H3K4me2/3 is also enhanced by H3Q5 serotonylation (Fig. 1C and D). Despite minor differences in *K*_D_, all data generated by us and others reinforce the significance of the Taf3-PHD interaction with serotonylated H3Q5.

**Figure 6.**
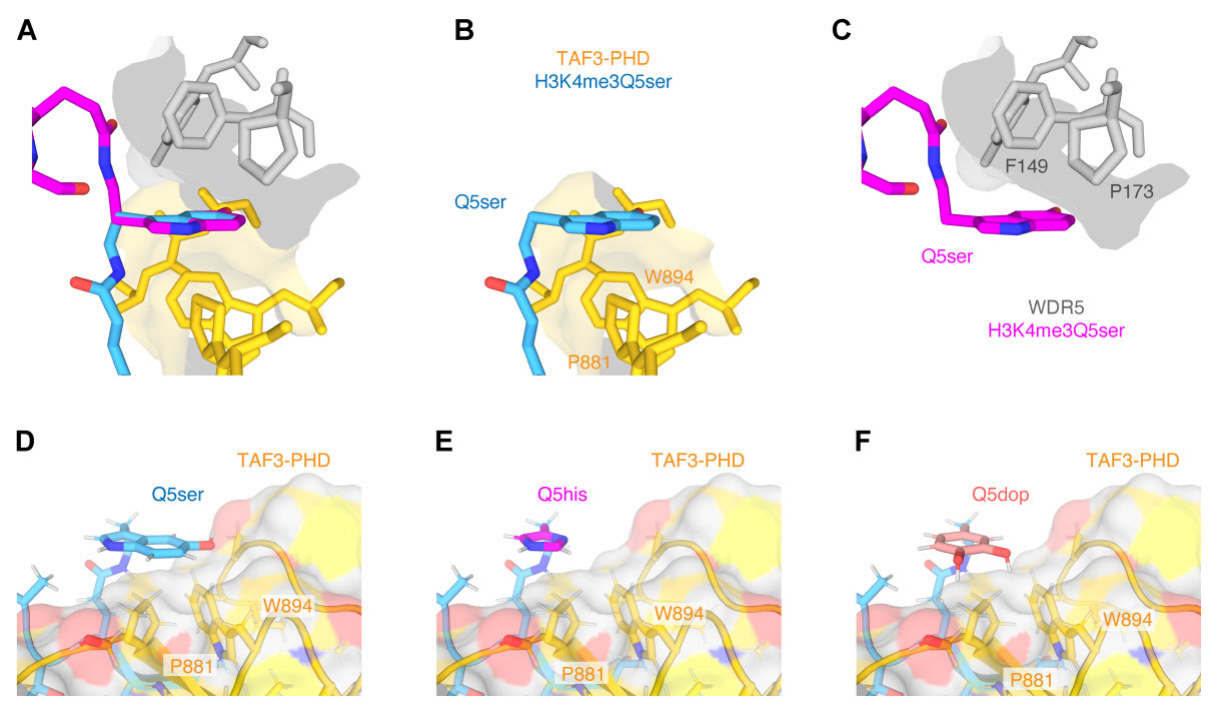
Observed and modelled edge–face interactions with H3Q5 monoaminylation modifications. (**A**) Comparison of H3Q5ser edge–face interaction with Taf3-PHD in panel (**B**) and WDR5 (PDB: 7CFP) in panel (**C**). Structure of H3Q5ser (**D**) compared to model of H3Q5his (**E**) and H3Q5dop (**F**) in complex with Taf3-PHD. Atomic coordinates for histamine and dopamine ligands (from the PDB) were superimposed on the serotonin aromatic in the H3K4me3Q5ser/Taf3-PHD complex. In this conformation, the histamine and serotonin rings stack exclusively on P881. Stacking on W894 would require conformational changes in the Q5 side chain and the his/dop linker to shift the position of the ring.

Importantly, the identification of an edge–face interaction mediated by P881 and stabilized by W894 as the key binding mode of serotonin to Taf3 provides an explanation to why H3Q5 enhances H3K4me3 engagement. The combination of a proline–tryptophan pair is unique to the PHD finger of TAF3 and conserved across TAF3 orthologues (Fig. 5B and Supplementary Figs S11 and S12). Studies of WDR5 describe an edge–face interaction, which relies on F149 of WDR5 as the proposed driver contact for H3Q5ser preference that would be supported by additional interactions such as a hydrogen bond with N130 [61, 62]. Strikingly, WDR5 contains a proline (P173) in the vicinity of F149 and H3Q5ser in a mirrored arrangement compared to the Taf3-PHD (Fig. 6A and C, and Supplementary Fig. S15B). Interestingly, in the Taf3-PHD structure H3Q5ser is mostly on top of the essential P881 with additional support from W894, while in WDR5 the H3Q5ser is mostly on top of F149 possibly with additional support from P173. Recently, the H3Q5ser interaction of WDR5 was characterized by its tunability towards the electron orbital distribution of the interacting residue [621]. This is further highlighted in the comparison of the WDR5 and TAF3 structures. In WDR5, the interacting groups are the polarized CH groups of F149, while in TAF3 these CH groups of P881 are less polarized. It is notable that changing electron orbital distribution of the serotonin has a negative effect on affinity [23].

Overall, we propose that serotonin recognition is governed by edge–face CH–π interactions with either aromatic residues or prolines. Consistent with our proposal, CHD1 contains aromatic residues at positions Y295 and Y406, either of which could orient to engage with H3Q5ser explaining the preference of CHD1 for serotonin (Supplementary Fig. S15C) [64]. Similarly, in SPIN1 residues F94 and/or G93, with similar exposed CH_2_ group as proline, could interact with H3Q5ser in an edge-faced manner (Supplementary Fig. S15D) [65]. Taking these observations into account, it is tempting to propose comparable edge–face binding modes for other monoaminylation modifications of H3Q5. TAF3-PHD displays a two- to three-fold increase in affinity for H3Q5his compared to unmodified H3 [13], consistent with a binding mode similar to the one described here for H3Q5ser leading to a similar effect on affinity. Indeed, both histaminylation (Fig. 6E) and dopaminylation (Fig. 6F) of H3Q5 can be modelled in edge–face interactions with Taf3-PHD. Due to their smaller aromatic rings compared to serotonin, histamine and dopamine could stack either on P881 or with some conformational adjustments on W894. These models will be useful in future studies towards the molecular details of histamine- or dopamine-modified chromatin.

Despite its recent discovery, histone serotonylation is being linked to multiple processes, which are essential for proper development, adaptation to the environment, and disease progression [11, 15–22]. Of particular interest is carcinoid syndrome (CS), which is associated with neuroendocrine tumours of the gut [66]. Excess serotonin production by these tumours has been linked to CS-associated diarrhoea and fibrosis [67]. However, it is currently unclear whether this relates to endocrine dysfunction or to increased histone H3Q5ser levels. By mutation of TAF3 residues P881 and W894, the endocrine and epigenetic effects of serotonin can be decoupled. Thus, the precise understanding of H3Q5ser recognition by the TAF3-PHD forms the foundation to decipher physiological functions of H3Q5ser and of other H3Q5 monoaminylations.

## Supplementary Material

gkaf612_Supplemental_File

## Data Availability

The mass spectrometry data underlying this article have been submitted to the PRIDE partner repository at https://www.ebi.ac.uk/pride/ under the dataset identifier PXD062109. The structure of TAF3-PHD in complex with H3K4me3Q5ser has been deposited to the PDB database (https://www.rcsb.org/) and can be accessed with PDB ID 9QLM. The NMR data are deposited to the BMRB database (https://bmrb.io/), under entry ID 34986.
