# Supplementary material for: Molecular determinants for recognition of serotonylated chromatin
Source: Nucleic Acids Res. 2025 Jul 9;53(13):gkaf612. doi: 10.1093/nar/gkaf612 (PMC12238850; doi:10.1093/nar/gkaf612)
Supplement: gkaf612_Supplemental_File [file gkaf612_supplemental_file.docx]

| **Table S1.** Structural statistics for the Taf3-PHD/H3K4me3Q5ser complex | | |
| --- | --- | --- |
| *A. Restraint information* |  |  |
| Total number of experimental distance restraints | 405 | |
| PHD domain  intra-residual/sequential/medium/long | 141 51/42/16/32 | |
| H3 peptide  intra-residual/sequential/medium/long | 155  97/42/16/0 | |
| Intermolecular restraints  A1/R2/T3/K4me3/Q5ser/T6/A7  q | 109  18/4/26/28/32/3/2 | |
| *B. Average deviation from experimental restraints* | | |
| All experimental distance restraints (Å) | 0.026 ± 0.003 | |
| Average number of violations > 0.5 Å | 0 | |
| *C. Coordinate RMS deviation (Å)* |  | |
| *Average overall RMSD to mean structure*^a^ |  | |
| Ordered heavy backbone atoms | 0.69 ± 0.11  0.49 | |
| Ordered all heavy atoms | 0.98 ± 0.11  0.82 | |
| Global backbone atoms | 1.91 ± 0.39  1.64 | |
| Global all heavy atoms | 2.47 ± 0.35  2.25 | |
| *H3K4me3Q5ser ligand RSMD to mean structure^b^* |  | |
| Ordered heavy backbone atoms | 0.72 ± 0.12  0.70 ± 0.15 | |
| Ordered all heavy atoms | 1.27 ± 0.10  1.19 ± 0.16 | |
| Q5ser all heavy atoms | 0.77 ± 0.29 | |
| Global heavy backbone atoms | 3.68 ± 1.05  2.35 ± 0.61 | |
| Global all heavy atoms | 3.05 ± 0.97  3.23 ± 0.64 | |
| *D. Ramachandran plot quality parameters (%)^a^* |  |  |
| Residues in most favoured regions (ordered/global) | 81.1 / 72.8  83.0 /± 4.5 | |
| Residues in allowed regions (ordered/global) | 17.4 / 23.2  13.5 ± 4.0 | |
| Residues in additionally allowed regions (ordered/global) | 1.0 / 2.6  2.3 ± 2.1 | |
| Residues in disallowed regions (ordered/global) | 0.5 / 1.4  1.1 ± 1.1 | |
| *E. Abnormalities found in structural checks* |  | 0 |
| Abnormally short interatomic distances | 0 ± 2 | |
| *F. RMSD to H3K4me3 complex lowest energy structure (Å)^c^* | | |
| TAF3-PHD ordered backbone | 0.98  0.79 ± 0.02 | |
| TAF3-PHD ordered heavy atom | 1.55  0.72 ± 0.03 | |
| H3K4me3 ordered backbone | 1.14  0.68 ± 0.10 | |
| H3K4me3 ordered heavy atom | 1.53  0.83 ± 0.08 | |
| ^a^ statistics are given for residues 857-924 of TAF3-PHD and residues 1-12 of H3K4me3Q5ser. Ordered regions are residues 857-918 of TAF3-PHD and residues 1-5 of H3K4me3Q5ser.  ^b^ RMSD for peptide (ligand) atoms after superposition of TAF3-PHD on all ordered heavy atoms  ^c^ using most-representative structure H3K4me3Q5ser complex | | |

**SUPPLEMENTARY DATA**

**Supplementary Table 1:** Structural statistics for the Taf3-PHD/H3K4me3Q5ser complex.

RESIdue QSR ! Added by Rodrigo Honorato / Alexandre Bonvin 06/01/2021

group

ATOM N TYPE=NH1 CHARge=-0.570 end

ATOM HN TYPE=H CHARge= 0.370 end

ATOM CA TYPE=CH1E CHARge= 0.200 end

ATOM HA TYPE=HA CHARge= 0.000 end

ATOM CB TYPE=CH2E CHARge= 0.000 end

ATOM HB1 TYPE=HA CHARge= 0.000 end

ATOM HB2 TYPE=HA CHARge= 0.000 end

ATOM CG TYPE=CH2E CHARge= 0.000 end

ATOM HG1 TYPE=HA CHARge= 0.000 end

ATOM HG2 TYPE=HA CHARge= 0.000 end

ATOM CD TYPE=C CHARge= 0.500 end

ATOM OE1 TYPE=O CHARge=-0.500 end

ATOM NE2 TYPE=NH1 CHARge=-0.570 end

ATOM HE2 TYPE=H CHARge= 0.370 end

ATOM CSA TYPE=CH2E CHARge= 0.200 end

ATOM HSA1 TYPE=HA CHARge= 0.000 end

ATOM HSA2 TYPE=HA CHARge= 0.000 end

ATOM CSB TYPE=CH2E CHARge= 0.000 end

ATOM HSB1 TYPE=HA CHARge= 0.000 end

ATOM HSB2 TYPE=HA CHARge= 0.000 end

! serotonin

ATOM HSE1 TYPE=H CHARge= 0.420 end

ATOM NSE1 TYPE=NH1 CHARge=-0.570 end

ATOM CSD1 TYPE=CR1E CHARge= 0.130 end

ATOM HSD1 TYPE=HA CHARge= 0.000 end

ATOM CSG TYPE=C5W CHARge=-0.055 end

ATOM CSD2 TYPE=CW CHARge=-0.055 exclude=(CSH2) end

ATOM CSE2 TYPE=CW CHARge= 0.130 exclude=(CSZ3) end

ATOM CSE3 TYPE=CR1E CHARge= 0.000 exclude=(CSZ2) end

ATOM HSE3 TYPE=HA CHARge= 0.000 end

ATOM CSZ3 TYPE=CY2 CHARge= 0.265 exclude=(CSE2) end

ATOM OSZ3 TYPE=OH1 CHARge=-0.700 end

ATOM HSZ3 TYPE=H CHARge= 0.435 end

ATOM CSH2 TYPE=CR1W CHARge= 0.000 exclude=(CSE2) end

ATOM HSH2 TYPE=HA CHARge= 0.000 end

ATOM CSZ2 TYPE=CR1W CHARge= 0.000 exclude=(CSE3) end

ATOM HSZ2 TYPE=HA CHARge= 0.000 end

!

ATOM C TYPE=C CHARge= 0.500 end

ATOM O TYPE=O CHARge=-0.500 end

BOND N HN

BOND N CA

BOND CA C BOND CA HA

BOND CA CB

BOND CB CG BOND CB HB1 BOND CB HB2

BOND CG CD BOND CG HG1 BOND CG HG2

BOND CD OE1 BOND CD NE2

BOND NE2 CSA BOND NE2 HE2

BOND CSA CSB BOND CSA HSA1 BOND CSA HSA2

BOND CSB CSG BOND CSB HSB1 BOND CSB HSB2

BOND CSG CSD2 BOND CSG CSD1

BOND CSD2 CSE2 BOND CSD2 CSE3

BOND CSE2 NSE1 BOND CSE2 CSZ2

BOND CSZ2 CSH2 BOND CSZ2 HSZ2

BOND CSH2 CSZ3 BOND CSH2 HSH2

BOND CSZ3 CSE3 BOND CSZ3 OSZ3 BOND OSZ3 HSZ3

BOND CSE3 HSE3

BOND NSE1 CSD1 BOND NSE1 HSE1

BOND CSD1 HSD1

BOND C O

ANGLe HSE1 NSE1 CSD1

ANGLe HSE1 NSE1 CSE2

ANGLe CSD1 NSE1 CSE2

ANGLe NSE1 CSD1 HSD1

ANGLe NSE1 CSD1 CSG

ANGLe HSD1 CSD1 CSG

ANGLe CSD1 CSG CSB

ANGLe CSD1 CSG CSD2

ANGLe CSB CSG CSD2

ANGLe CSG CSB HSB2

ANGLe CSG CSB HSB1

ANGLe CSG CSB CSA

ANGLe HSB2 CSB HSB1

ANGLe HSB2 CSB CSA

ANGLe HSB1 CSB CSA

ANGLe CSB CSA HSA2

ANGLe CSB CSA HSA1

ANGLe CSB CSA NE2

ANGLe HSA2 CSA HSA1

ANGLe HSA2 CSA NE2

ANGLe HSA1 CSA NE2

ANGLe CSA NE2 HE2

ANGLe CSG CSD2 CSE3

ANGLe CSG CSD2 CSE2

ANGLe CSE3 CSD2 CSE2

ANGLe CSD2 CSE3 HSE3

ANGLe CSD2 CSE3 CSZ3

ANGLe HSE3 CSE3 CSZ3

ANGLe CSE3 CSZ3 OSZ3

ANGLe CSE3 CSZ3 CSH2

ANGLe OSZ3 CSZ3 CSH2

ANGLe CSZ3 OSZ3 HO1

ANGLe CSZ3 CSH2 HSH2

ANGLe CSZ3 CSH2 CSZ2

ANGLe HSH2 CSH2 CSZ2

ANGLe CSH2 CSZ2 HSZ2

ANGLe CSH2 CSZ2 CSE2

ANGLe HSZ2 CSZ2 CSE2

ANGLe NSE1 CSE2 CSD2

ANGLe NSE1 CSE2 CSZ2

ANGLe CSD2 CSE2 CSZ2

! around the 6-ring

IMPRoper CSD2 CSE2 CSZ2 CSH2

IMPRoper CSE2 CSZ2 CSH2 CSZ3

IMPRoper CSZ2 CSH2 CSZ3 CSE3

IMPRoper CSH2 CSZ3 CSE3 CSD2

IMPRoper CSZ3 CSE3 CSD2 CSE2

IMPRoper CSE3 CSD2 CSE2 CSZ2

! link 5-ring to 6-ring

IMPRoper CSD1 NSE1 CSE2 CSZ2

IMPRoper CSD1 CSG CSD2 CSE3

IMPRoper NSE1 CSE2 CSZ2 CSH2

IMPRoper NSE1 CSE2 CSD2 CSE3

IMPRoper CSG CSD2 CSE3 CSZ3

IMPRoper CSG CSD2 CSE2 CSZ2

! 6-ring hydrogens

IMPRoper HSZ2 CSZ2 CSH2 CSZ3

IMPRoper HSH2 CSH2 CSZ3 CSE3

IMPRoper OSZ3 CSZ3 CSH2 CSZ2

IMPRoper HSE3 CSE3 CSZ3 CSH2

! 5-ring hydrogens and CB

IMPRoper HSE1 NSE1 CSE2 CSD2

IMPRoper HSD1 CSD1 NSE1 CSE2

IMPRoper CSB CSG CSD2 CSE2

! planarity of side-chain CO-N BOND

IMPRoper CD CG OE1 NE2 ! planar -C

IMPRoper NE2 CD CSA HE2 ! planar +N

IMPRoper CG CD NE2 HE2 ! ANGLe across peptide plane

IMPRoper CG CD NE2 CSA ! ANGLe across peptide plane

!

IMPRoper HA N C CB !chirality CA

IMPRoper HB1 HB2 CA CG !stereo CB

IMPRoper HG2 HG1 CB CD !stereo CG

IMPRoper HSA1 HSA2 NE2 CSB !stereo CSA

IMPRoper HSB1 HSB2 CSA CSG !stereo CSB

DIHEdral CSB CSA NE2 HE2

DIHEdral CSG CSB CSA NE2

DIHEdral CSD2 CSG CSB CSA

DIHEdral CSH2 CSZ3 OSZ3 HSZ3

DIHEdral CG CB CA N

DIHEdral CD CG CB CA

DIHEdral OE1 CD CG CB

DONO HN N

DONO HE2 NE2

DONO HSE1 NSE1

DONO HSZ3 OSZ3

ACCE OSZ3 CSZ3

ACCE OE1 CD

ACCE O C

END

**Supplementary Table 2:** CNS Topology definitions for QSR residue as implemented in HADDOCK.


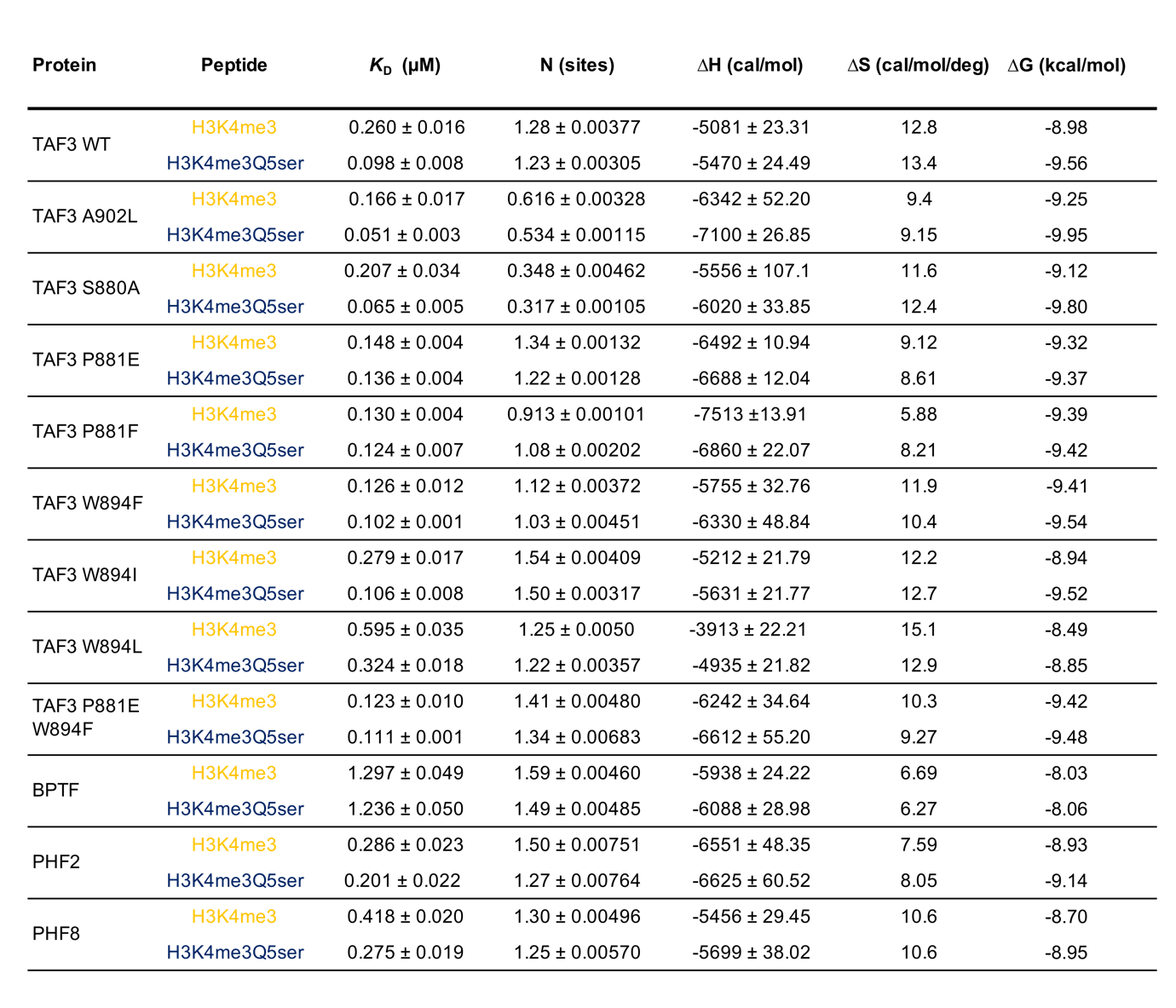


**Supplementary Table 3:** ITC analysis data. Curve fitting analysis was performed following the one-set-of-sites model in the MicroCal-ITC Origin 7 software.

**Supplementary Figure 1:** SDS-PAGE followed by Coomassie staining of all purified protein samples used. (**A**) GST-tagged proteins used in peptide pull-down followed by SDS-PAGE and ITC. (**B**) and (**C**) ^13^C and ^15^N isotopically labelled Taf3-PHD WT and double mutant, respectively.

**A** H-ARTK(Me_3_)Q(ser)TARKSTGGK(biotin)-NH_2_

Calculated for C_83_H_142_N_27_O_23_S^+^: 1916.0647, found: XXXX [M]^+^

**B** H-ARTK(Me_2_)Q(ser)TARKSTGGK(biotin)-NH_2_

Calculated for C_82_H_141_N_28_O_22_S^+^: 1902.0490, found: XXXX [M+H]^+^

**C** H-ARTK(Me)Q(ser)TARKSTGGK(biotin)-NH_2_

Calculated for C_81_H_139_N_28_O_22_S^+^: 1888.0334, found: XXXX [M+H]^+^

**D** H-ARTKQ(ser)TARKSTGGK(biotin)-NH_2_

Calculated for C_80_H_137_N_28_O_22_S^+^: 1874.0177, found: XXXX [M+H]^+^

**E** H-ARTKQTARKSTGGK(biotin)-NH_2_

Calculated for C_70_H_128_N_27_O_21_S^+^: 1714.9493, found: XXXX [M+H]^+^

(no 280 nm chromophore)

**F** H-ARTK(Me_3_)QTARKSTGGK(biotin)-NH_2_

Calculated for C_73_H_134_N_27_O_21_S^+^: 1756.9963, found: XXXX [M]^+^

(no 280 nm chromophore)

**G** H-ARTK(Me_2_)QTARKSTGGK(biotin)-NH_2_

Calculated for C_72_H_132_N_27_O_21_S^+^: 1742.9806, found: XXXX [M+H]^+^

(no 280 nm chromophore)

**H** H-ARTK(Me)QTARKSTGGK(biotin)-NH_2_

Calculated for C_71_H_130_N_27_O_21_S^+^: 1728.9650, found: XXXX [M+H]^+^

(no 280 nm chromophore)

**I** H-ARTK(Me_3_)Q(ser)TARKSTG-NH_2_

Calculated for C_65_H_114_N_23_O_18_^+^: 1504.8707, found: XXXX [M]^+^

**J** H-ARTK(Me_2_)Q(ser)TARKSTG-NH_2_

Calculated for C_64_H_112_N_23_O_18_^+^: 1490.8550, found: XXXX [M+H]^+^

**K** H-ARTK(Me)Q(ser)TARKSTG-NH_2_

Calculated for C_63_H_110_N_23_O_18_^+^: 1476.8394, found: XXXX [M+H]^+^

**L** H-ARTKQ(ser)TARKSTG-NH_2_

Calculated for C_62_H_108_N_23_O_18_^+^: 1462.8237, found: XXXX [M+H]^+^

**M** H-ARTKQTARKSTG-NH_2_

Calculated for C_52_H_99_N_22_O_17_^+^: 1303.7553, found: XXXX [M+H]^+^

(no 280 nm chromophore)

**N** H-ARTK(Me_3_)QTARKSTG-NH_2_

Calculated for C_55_H_105_N_22_O_17_^+^: 1345.8023, found: XXXX [M]^+^

(no 280 nm chromophore)

**O** H-ARTK(Me_2_)QTARKSTG-NH_2_

Calculated for C_54_H_103_N_22_O_17_^+^: 1331.7866, found: XXXX [M+H]^+^

(no 280 nm chromophore)

**P** H-ARTK(Me)QTARKSTG-NH_2_

Calculated for C_53_H_101_N_22_O_17_^+^: 1317.7710, found: XXXX [M+H]^+^

(no 280 nm chromophore)

**Supplementary Figure 2:** Characterisation details for synthetic peptides.

**Supplementary Figure 3:** (**A**, **B**) Structure of serotonylated glutamine. Panel (**B**) shows the 3D structure as implemented in HADDOCK as residue QSR with atom naming HSX for hydrogen atoms in the serotonin group, following the conventional atom names of tryptophan. (**C**) ^1^H 1D spectrum of the non-biotinylated H3K4me3Q5ser peptide in NMR buffer with assignments indicated. Peaks labelled * are from minor impurities.

**Supplementary Figure 4:** H3Q5ser potentiates recombinant GST-Taf3-PHD binding to H3 histone tails. (**A**) Histone peptide pull-down using bacterial lysate overexpressing GST-Taf3-PHD fusion. Comparisons were made between non-methylated as well as H3K4 mono-, di- and tri-methylated peptides and their Q5 serotonylated counterparts. Bound proteins were resolved by SDS-PAGE and stained with Coomassie. The GST-Taf3 position is indicated with an arrow. Relative band intensity was analysed using the Image Lab 6.1 software (Bio-Rad) and is indicated on the bottom of the figure.


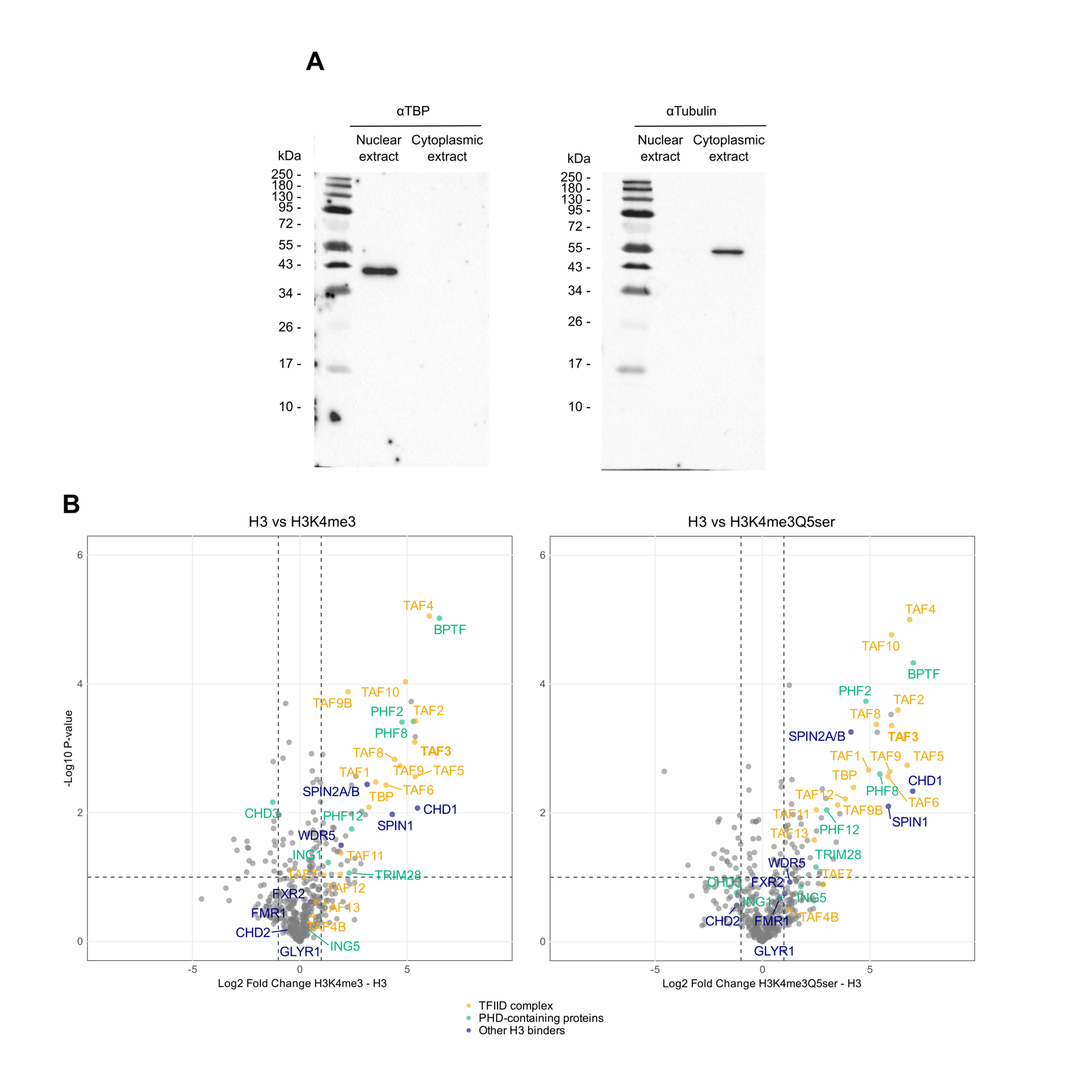


**Supplementary Figure 5:** (**A**) Immunoblot validation of cell fractionation for Peptide Pull-Down followed by Mass Spectrometry. (**B**) Mass Spectrometry data comparing unmodified H3 to H3K4me3 in presence (right) or absence (left) of H3Q5 serotonylation. False discovery rate (FDR) cutoff is 0.1. Colour coding of identified proteins is indicated in the figure bottom.


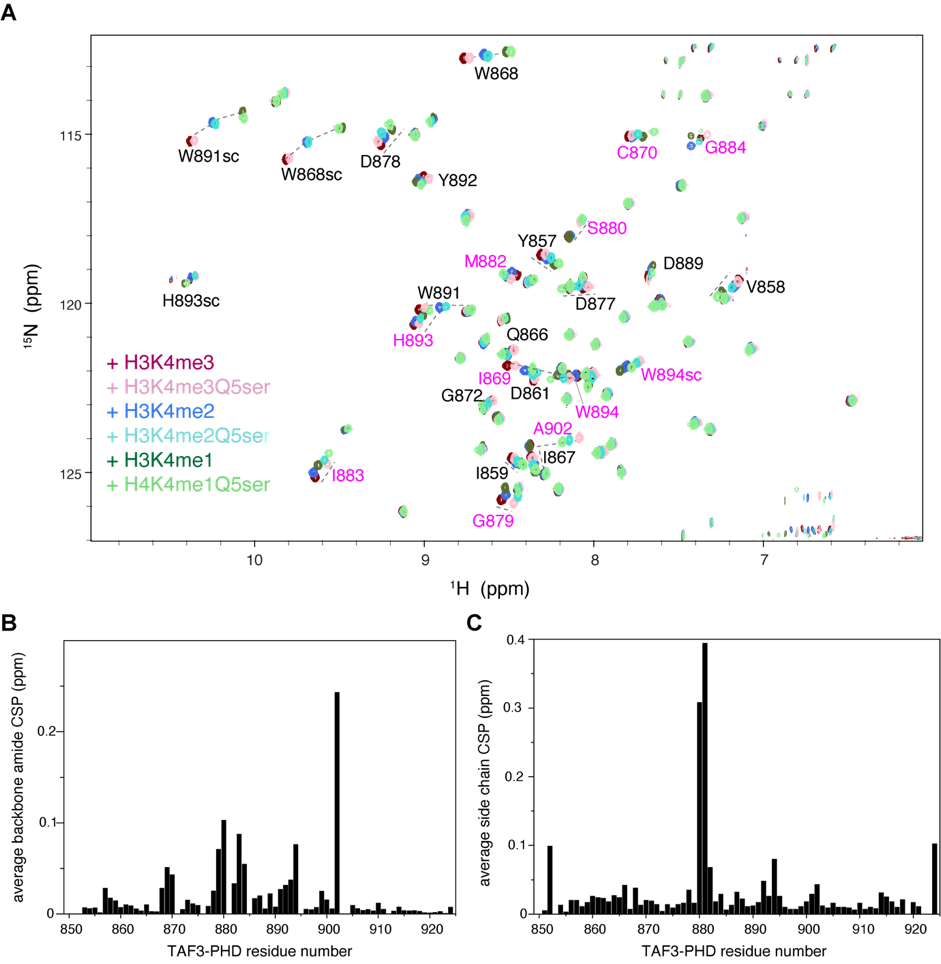


**Supplementary Figure 6:** (**A**) Overlay of ^15^N-HSQC spectra of Taf3-PHD with excess of indicated peptides. Resonances with significant CSPs are labelled and connected by dashed lines. Residues with H3Q5ser-dependent chemical shifts are indicated with magenta labels. (**B, C**) Average weighted amide backbone (**B**) and side chain (**C**) chemical shift perturbation per residue between the H3K4me3 and H3K4me3Q5ser complex.


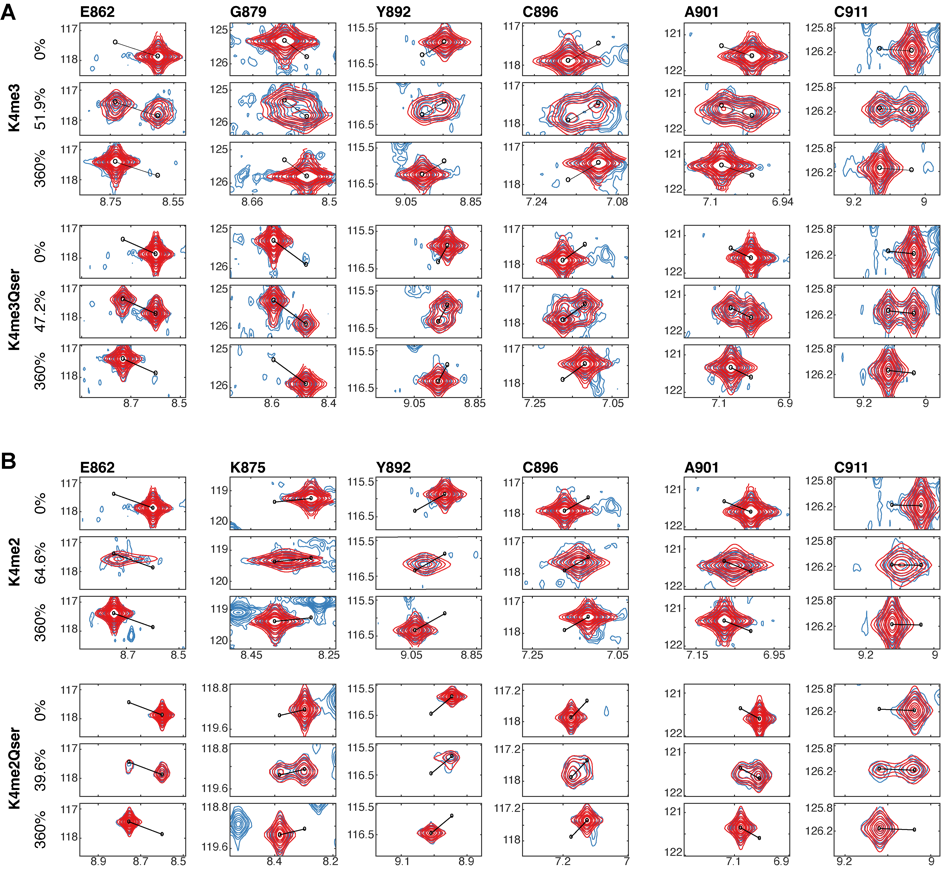


**Supplementary Figure 7:** Estimation of binding off-rate *k*_off_ from line shape in midpoint spectra. Experimental and fitted of free, ~50% bound and fully bound TAF3-PHD resonances for H3K4me3/H3K4me3Q5ser (**A**) and H3K4me2/H3K4me2Q5ser (**B**) complex. Experimental spectra in blue, fitted spectra in red. The exact amount of peptide added for the midpoint spectrum has been fitted and is indicated.


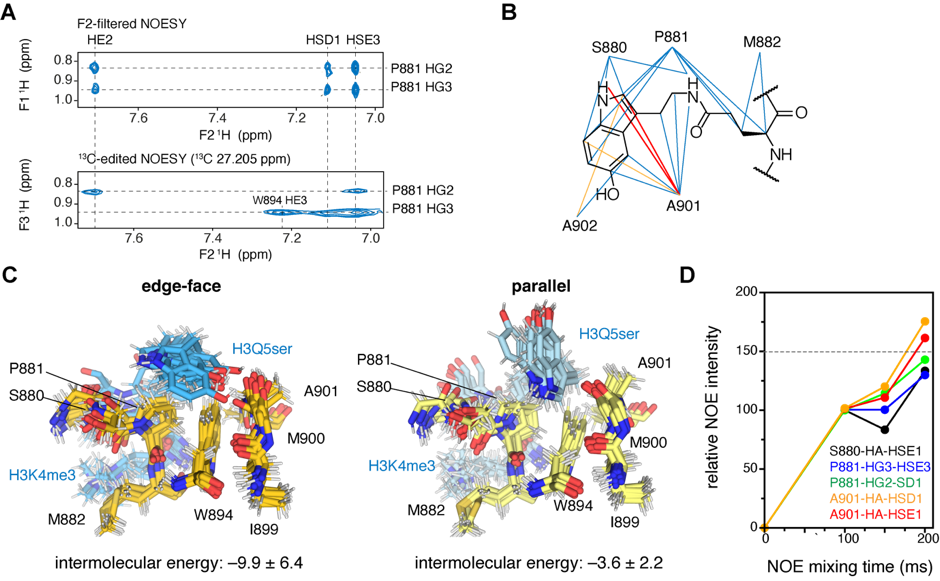


**Supplementary Figure 8:** Intermolecular NOEs defining the TAF3-PHD–H3K4me3Q5ser complex structure. (**A**) Section of 2D F2-filtered NOESY (top) and 3D 13C-edited NOESY (bottom) showing intermolecular NOEs between Q5ser protons HE2, HSD1 and HSE3 to P881 HG protons in TAF3-PHD. Note that the 3D NOESY view is transposed (acquisition dimension vertical) to highlight the alignment of the NOE cross peaks with the 2D NOESY. (**B**) Schematic showing the observed NOE network involving Q5ser protons. NOEs are indicated as solid lines connecting the (hetero)atom in Q5ser to a Taf3-PHD residue. The two NOEs indicated in red (caused by spin diffusion) and the two NOEs indicated in orange (structurally inconsistent, and possibly spin diffusion) were excluded from the final structure calculation. (**C**) Superposition of Taf3-PHD/H3K4me3Q5ser structures, zoomed in on the Q5ser residue, showing the two conformations obtained when including all intermolecular NOE data. When in the edge-face conformation, H3Q5ser has more extensive and favourable interactions with Taf3-PHD than in the parallel orientation, as indicated by the lower intermolecular energy score (in kcal/mol) as calculated by HADDOCK. (**D**) Relative intensity of indicated intermolecular NOEs as function of NOE mixing time, normalized to 100 at 100 ms mixing time. The relative higher intensity of A901 HA to Qser HSD1/HSE1 at 200 ms mixing time is indicative of indirect magnetization transfer via spin-diffusion (ref). Dashed line added to guide the eye.


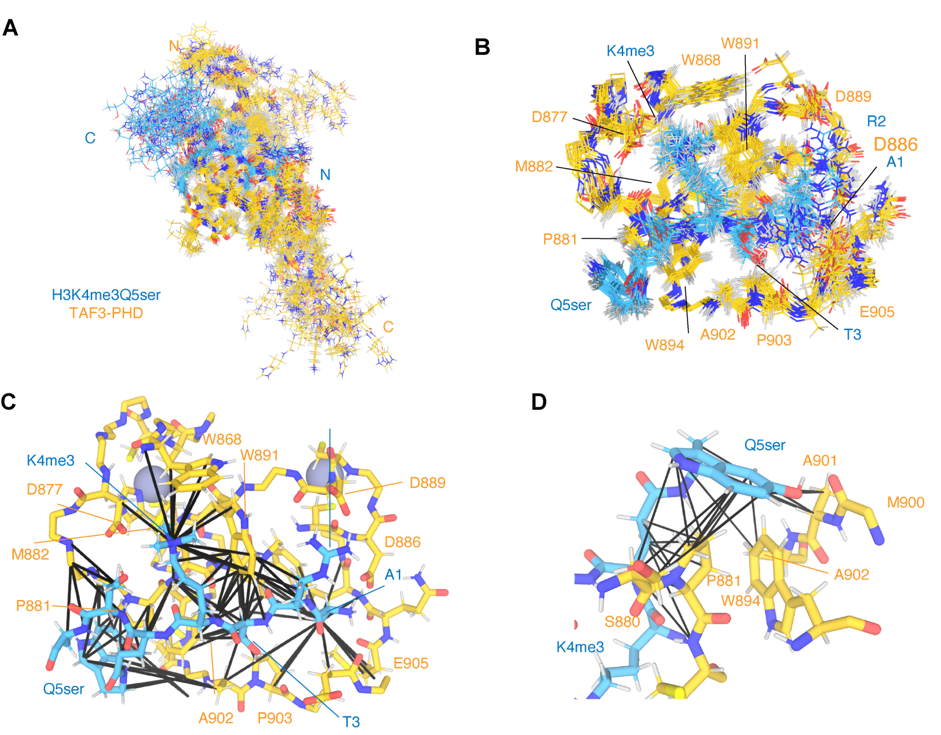


**Supplementary Figure 9:** (**A, B**) Superposition of the ensemble of 20 solution structures of the complex, showing the complete complex (**A**), and zoomed in on the peptide binding region (**B**). (**C**, **D**) Most representative structure of the complex, zoomed in on the peptide (**C**) or the Q5ser binding site (**D**) with intermolecular NOEs shown as solid dashed lines connecting the heteroatoms.


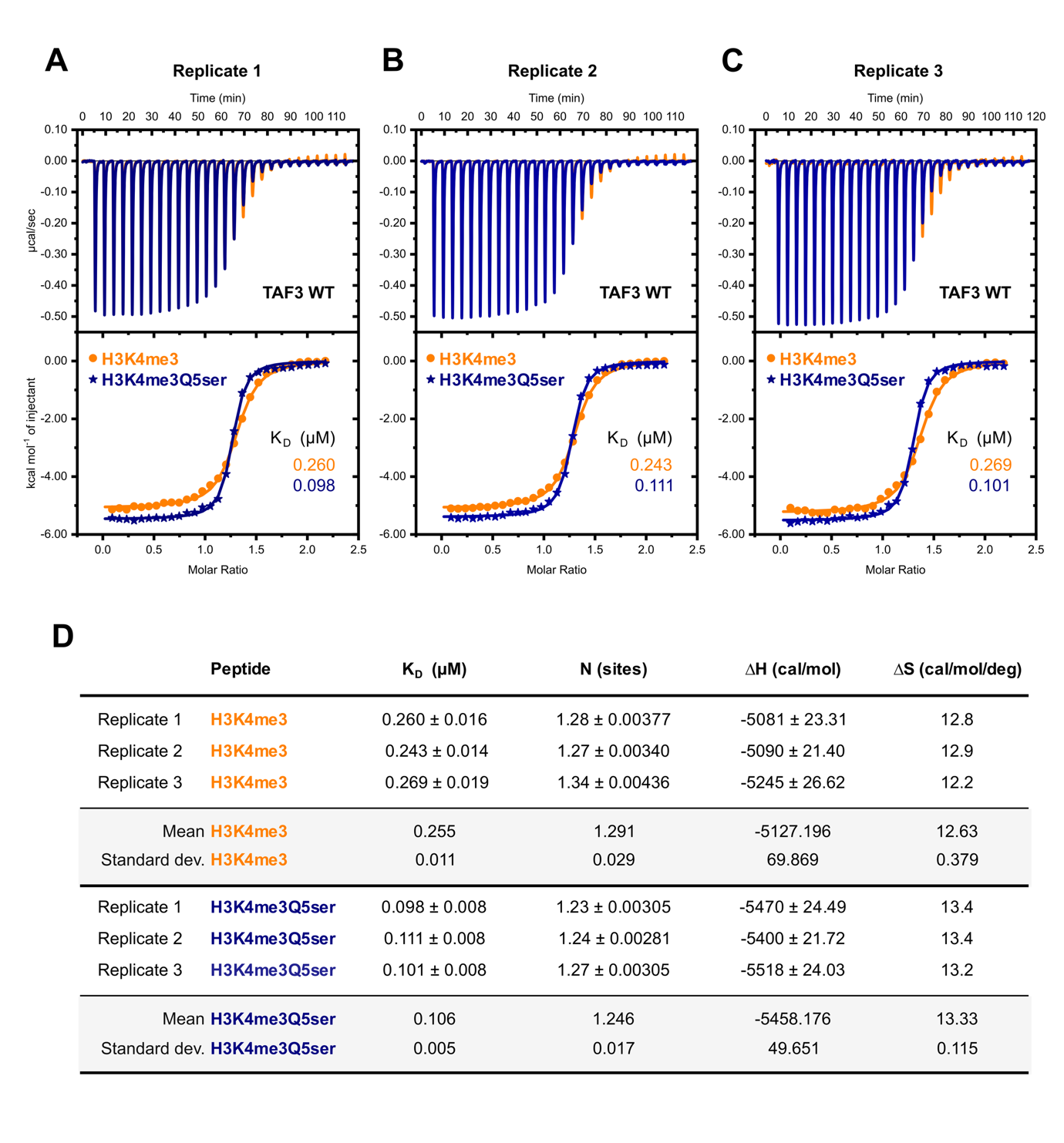


**Supplementary Figure 10:** Reproducibility of ITC results of H3K4me3 (orange curves) and of H3K4me3Q5ser (blue curves) peptide titrations with wild type TAF3-PHD fused to GST. Panels A to C represent three independent titrations performed over a timespan of five months. (D) Table of the corresponding ITC data as indicated above each column. Mean values and standard deviation of the triplicates are indicated at the bottom.

**Supplementary Figure 11:** Multiple sequence alignment of human PHD-containing proteins. Consensus Cys4-His-Cys3 positions are highlighted in grey. Positions corresponding to Taf3 P881 and W894 are highlighted in yellow (Figure on following page).


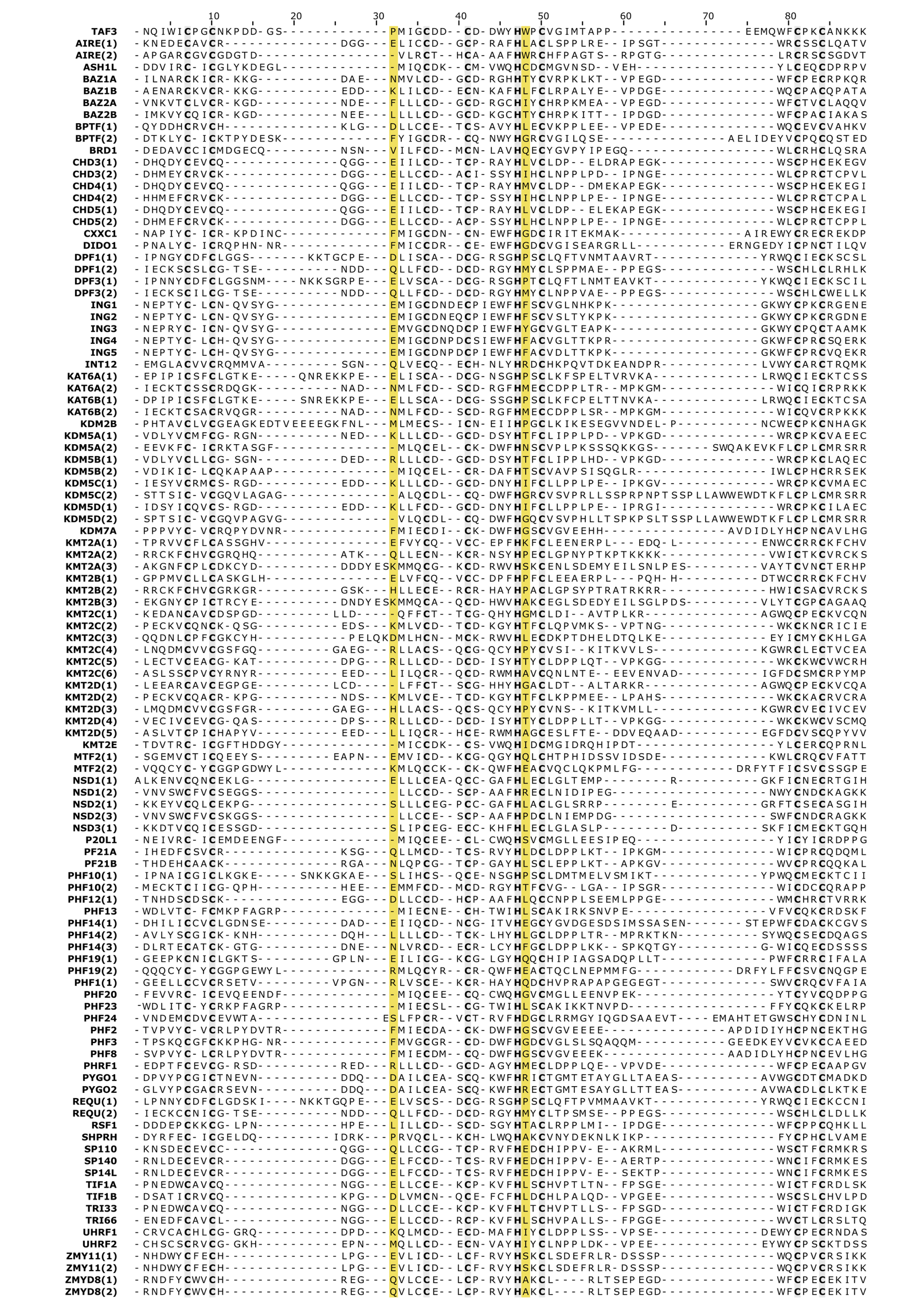


**
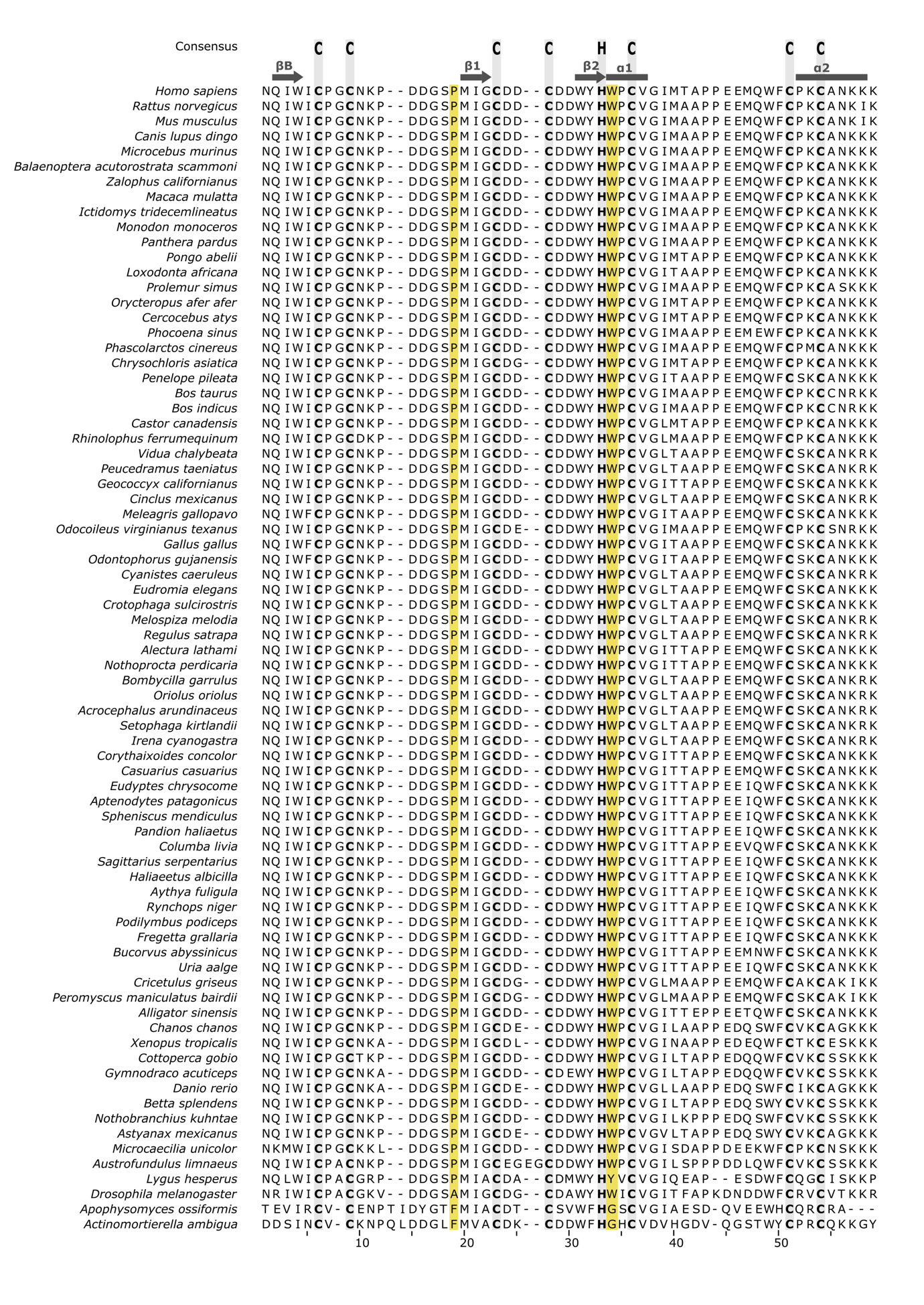
**

**Supplementary Figure 12:** Multiple sequence alignment of a selection of TAF3 orthologues PHD fingers across the indicated species. Consensus Cys4-His-Cys3 positions are highlighted in grey and indicated on top. Positions corresponding to Taf3 P881 and W894 are highlighted in yellow. Location of the secondary structure elements of TAF3-PHD are indicated on top (Figure on previous page).

**Supplementary Figure 13:** Characterization of P881E/W894F TAF3-PHD mutant. (**A**) 15N-HSQC fingerprint spectrum (50 μM, 600 MHz) with peak assignments indicated. Missing peaks are labelled in magenta at the corresponding peak position in wild-type TAF3-PHD. (**B**) Relative peak intensity in mutant (top) and weighted CSP between mutant and wild-type (bottom). (**C**) Structure of wild-type TAF3-PHD with two mutated residues (P881 and W894) indicated in red, residues with missing amide backbone peaks in yellow, and residues with high CSP (labelled red in (**B**)) labelled in orange. (**D**) Comparison of CSPs for wild-type (top) and mutant (bottom) upon binding serotonylated and non-serotonylated peptide. (*) indicates that peak is only observed in the bound-state. (*) indicates that peak is only observed in free state.

**Supplementary Figure 14:** (**A**, **B**) Experimental and fitted spectra of free, ~50% bound and fully bound P881E/W894F mutant TAF3-PHD resonances for H3K4me3 (**A**) and H3K4me3Q5ser (**B**) peptide. (**C**) Comparison of k_off_ values. Gray lines indicate 95% confidence limits.


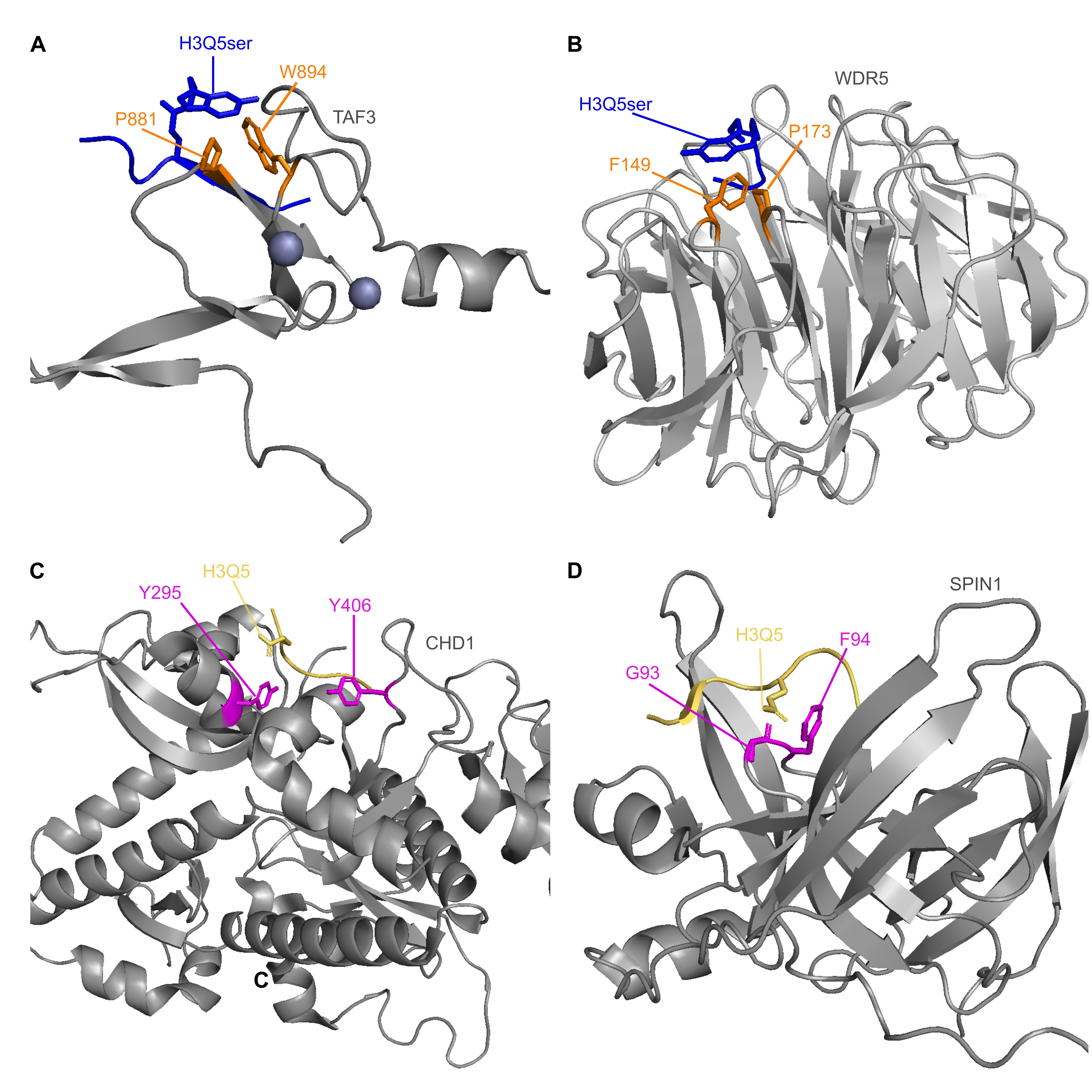


**Supplementary Figure 15:** Observed and predicted edge-face interactions with H3Q5ser in different H3 binding domains. (**A**) TAF3-PHD (in grey) residues P881 (in orange) direct edge-face interaction with H3K4me3Q5ser (in blue) while W894 (in orange) stabilises the interaction. (**B**) H3K4me3Q5ser (in blue) engaged with WDR5 (in grey) as an edge-face with residue F149 (in orange) and supported by P173 (in orange) based on PDB 7CFQ. (**C**) CHD1 (in grey) could interact with serotonylated H3Q5 (in yellow, serotonin not displayed) in an edge-face conformation with Y295 or Y406 (both in magenta), based on PDB 2B2V. (**D**) SPIN1 (in grey) could interact with serotonylated H3Q5 (in yellow, serotonin not displayed) in an edge-face conformation with F94 and/or G93 (in magenta) based on PDB 4H75.
